# *KCNJ15*/Kir4.2 couples with polyamines to sense weak extracellular electric fields in galvanotaxis

**DOI:** 10.1038/ncomms9532

**Published:** 2015-10-09

**Authors:** Ken-ichi Nakajima, Kan Zhu, Yao-Hui Sun, Bence Hegyi, Qunli Zeng, Christopher J. Murphy, J. Victor Small, Ye Chen-Izu, Yoshihiro Izumiya, Josef M. Penninger, Min Zhao

**Affiliations:** 1 Department of Dermatology, Institute for Regenerative Cures, School of Medicine, University of California Davis, Sacramento, California 95817, USA; 2 Bioelectromagnetics Laboratory, Zhejiang University School of Medicine, Hangzhou, Zhejiang 310058, China; 3 Department of Pharmacology, University of California Davis, Davis, California 95616, USA; 4 Department of Surgical and Radiological Sciences, School of Veterinary Medicine, University of California Davis, Davis, California 95616, USA; 5 Department of Ophthalmology & Vision Science, School of Medicine, University of California Davis, Sacramento, California 95817, USA; 6 IMBA, Institute of Molecular Biotechnology of the Austrian Academy of Sciences, 1030 Vienna, Austria; 7 Department of Biochemistry and Molecular Medicine, University of California at Davis, Sacramento, California 95616, USA

## Abstract

Weak electric fields guide cell migration, known as galvanotaxis/electrotaxis. The sensor(s) cells use to detect the fields remain elusive. Here we perform a large-scale screen using an RNAi library targeting ion transporters in human cells. We identify 18 genes that show either defective or increased galvanotaxis after knockdown. Knockdown of the *KCNJ15* gene (encoding inwardly rectifying K^+^ channel Kir4.2) specifically abolishes galvanotaxis, without affecting basal motility and directional migration in a monolayer scratch assay. Depletion of cytoplasmic polyamines, highly positively charged small molecules that regulate Kir4.2 function, completely inhibits galvanotaxis, whereas increase of intracellular polyamines enhances galvanotaxis in a Kir4.2-dependent manner. Expression of a polyamine-binding defective mutant of *KCNJ15* significantly decreases galvanotaxis. Knockdown or inhibition of *KCNJ15* prevents phosphatidylinositol 3,4,5-triphosphate (PIP_3_) from distributing to the leading edge. Taken together these data suggest a previously unknown two-molecule sensing mechanism in which *KCNJ15*/Kir4.2 couples with polyamines in sensing weak electric fields.

## Abstract (web summary)

Directed cell migration in weak electric fields is known as galvanotaxis, but the cellular sensor and mechanism is not known. Here Nakajima *et al.* identify inwardly rectifying K^+^ channel Kir4.2 as an important mediator of galvanotaxis, that depends on the cytoplasmic distribution of intracellular polyamines.

## Graphical abstract

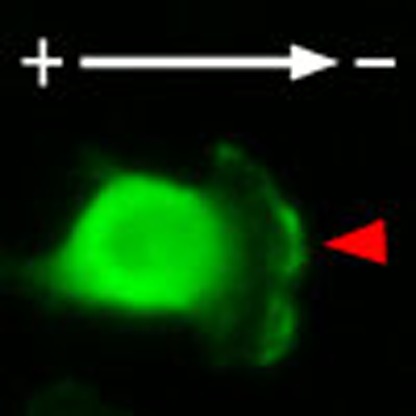

Being able to sense the environment is one of the most critical functions of living cells. Ligand–receptor binding is a well-understood biochemical sensing (signalling) mechanism. Cells are also responsive to mechanical force, electrical fields (EFs), heat and light. The sensing mechanisms for those physical factors are different and unique, and are much less well understood. Small physiological direct current EFs are found in living organisms from plants to animals, for example at wounds, regeneration sites and tumours, and provide a strong guidance cue for directional cell migration, a phenomenon termed galvanotaxis/electrotaxis that was first demonstrated over 100 years ago^1^,^2^,^3^,^4^. Small applied EFs can regulate cell migration, proliferation, morphology, orientation, polarization and even the collective behaviours of large cell groups^5^,^6^. Galvanotaxis may play a crucial role in wound healing/regeneration and development^7^,^8^,^9^,^10^,^11^,^12^,^13^. Interest in using this powerful mechanism to engineer cells and tissues is growing stronger^14^. Electric stimulation has long being attempted and trialled in many diverse conditions such as chronic wounds^15^. The molecules serving as the ‘sensor(s)’ for the weak EFs, however, remain unknown.

Ion channels are the key molecules involved in production and sensing of biological electrical activities. Ion channels conduct ion(s) across the cell membrane and play important roles for the generation of action potential in various excitable cells, the maintenance of resting membrane potential in either excitable and non-excitable cells, and homoeostasis of ionic environments of the cell^16^,^17^,^18^,^19^. Ion channels are localized at the plasma membrane and are among the first groups of molecules exposed to extracellular EFs; this category of proteins therefore would be a promising candidate to be the sensor of weak extracellular EFs. We developed a large-scale systematic screen to determine roles for ion channels in sensing weak EFs in galvanotaxis of human cells.

## Results

### Implementation of screening strategy

**Figure 1:**
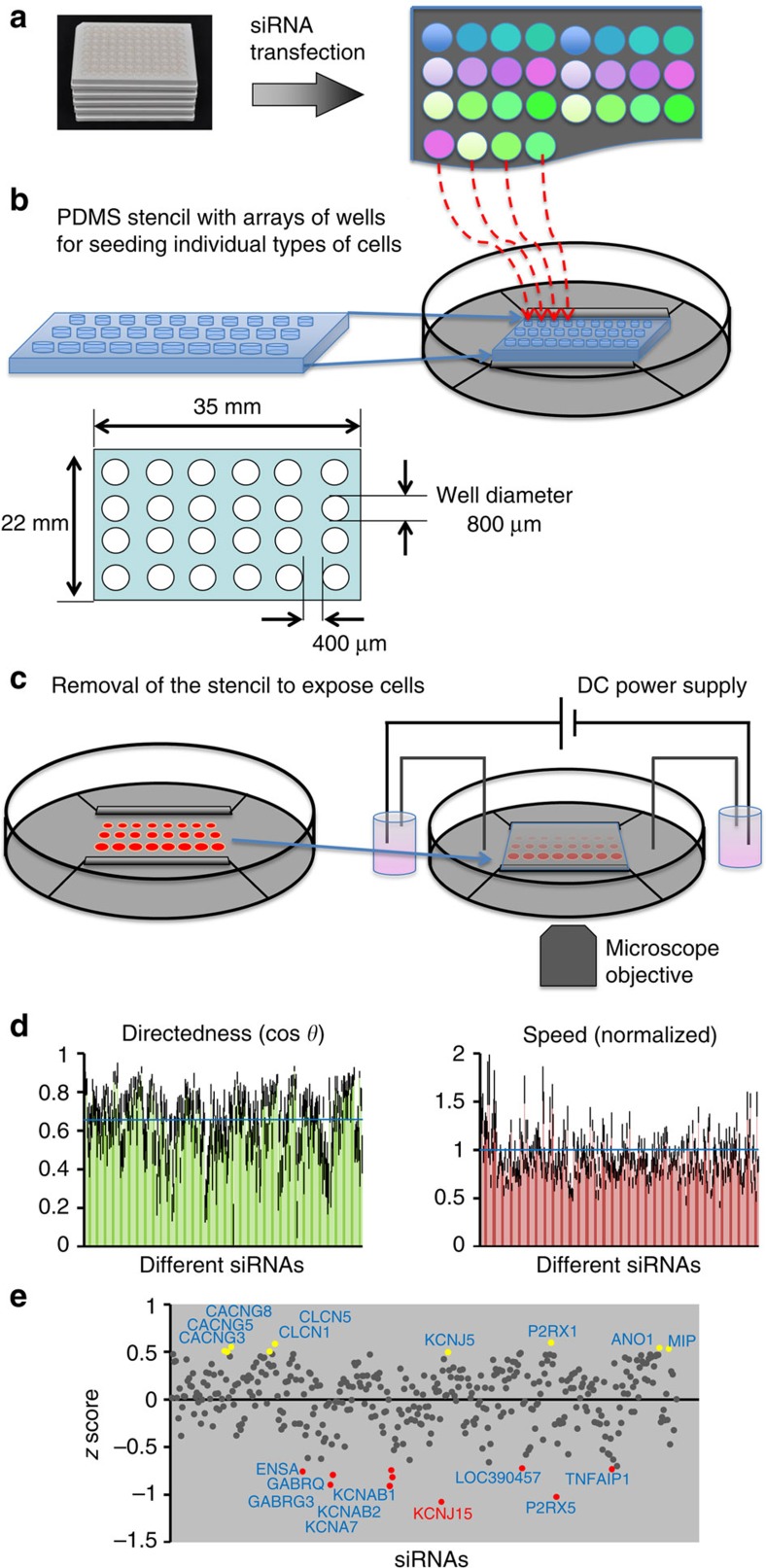
Large-scale RNAi screen for galvanotaxis phenotype. (**a**) hTCEpi cells transfected with siRNA from a library against human ion channels/pumps/transporters. (**b**) Polydimethylsiloxane (PDMS) stencil facilitated cell spotting in the galvanotaxis chamber. Cells, 48 h after transfection, were spotted onto galvanotaxis/electrotaxis chamber, pre-coated with FNC coating mixture, which could be guided by the stencil. (**c**) Multi-field video imaging to efficiently record cell migration of many types of cells in one experiment. After cells adhered to the culture dish, the stencil was removed. The chamber was covered with a coverslip. Direct current was applied. Cell migration was imaged with a time-lapse imaging system. (**d**) Knockdown of channels with the RNAi library revealed genes important for galvanotaxis. Graphs show migration directedness (cos *θ*) and migration speed from first screening of the whole library. Control is indicated by the blue line (cos *θ*=0.64). Migration speed was normalized to paired control (=1, indicated by blue line). (**e**) The screen identified genes critical for galvanotaxis. The *y* axis represents the *z* score of directedness (cos *θ*). Genes with *z* score >0.495 are highlighted in yellow, representing genes that after knockdown significantly increased galvanotaxis. Genes with *z* score <−0.7 are highlighted in red, representing genes that after knockdown significantly inhibited galvanotaxis. Cell numbers analysed for each conditions 35–69. EF=200 mV mm^−1^.

As a first step to discover the sensor molecule(s) for galvanotaxis, we developed large-scale screening methods to identify ion channel genes that are important in galvanotaxis. We used the On-target plus siRNA human ion channel siRNA library. This library contains 381 siRNAs against genes coding human ion channels, pumps and transporters (Fig. 1, Supplementary Fig. 1). We transfected siRNA individually into telomerase immortalized human corneal epithelial cells (hTCEpi cells) using Lipofectamine 2000 reagent. Transfection efficiency was over 95% as judged from fluorescence of control oligo transfection (Supplementary Fig. 1c).

We used multi-spot seeding to screen for the galvanotaxis phenotype in large numbers of different types of cells. To increase screen efficiency, we developed stencils with multi-wells in which cells after different treatments could be seeded separately. Placing the stencil on the culture dish allowed us to simultaneously seed cells on spot arrays. Cells after transfection with different siRNA can therefore be seeded separately on each bottomless well without cross contamination. We used polydimethylsiloxane materials that adhere to the culture dish base with a water-tight seal that prevents well to well exchange of medium or cells. Our current galvanotaxis chamber allows up to 50 different treatments. At 48 h after transfection cells were trypsinized and seeded into the wells of the galvanotaxis chamber pre-coated with FNC Coating Mix. After cells adhere to the dish, the stencil can be lifted and removed (Fig. 1b,c). The cells were then exposed to EFs. On a motorized stage with multi-field video imaging, cells transfected with different siRNAs on up to 50 different spots can be video imaged at the same time. Galvanotactic migration was recorded with an inverted microscope for 30 min in a direct current EF of 200 mV mm^−1^, and quantitatively analysed using ImageJ. This method increased screening efficiency 50 times or more compared with traditional galvanotaxis experiments. Importantly, cells transfected with different siRNAs were processed and imaged at the same time in the same chamber together with the transfection control, minimizing batch to batch variation and significantly optimizing comparability of migration analyses.

### RNAi screening identified genes important in galvanotaxis

We used the large-scale screening strategy to obtain galvanotaxis profiles after knockdown of individual ion channel subunits. We quantified directedness (cos *θ*) and migration speed using ImageJ software with MTrackJ and Chemotaxis Tool plugins (see Methods section). The directedness value quantifies how directionally the cells move in the field direction. Migration of a population of cells toward the cathode gives a directedness value larger than 0 and approaching 1, with a value of exactly 1 indicating a cell moved straight to the cathode. Migration of a population of cells towards the anode gives a directedness value smaller than 0 and approaching −1. Knockdown of some channels showed significant effects on both migration directedness and speed, while some affected migration directedness more than the speed, and some affected speed more than the directedness. Compilation of the directedness and speed data demonstrated the profiling of galvanotaxis after knockdown of individual ion channels in the library (Fig. 1d).

To identify which gene knockdowns showed significant effects on the migration speed and directedness, we set cutoff lines at 2.5% of the population distribution of both the directedness and migration values after knockdown. This analysis identified 35 gene knockdowns that showed significant effects on galvanotaxis. All except one affected migration speed or directedness separately, not both. After knockdown, 18 genes significantly affected directedness—*KCNJ15*, *KCNAB2* and 7 others genes significantly decreased the directedness value, while knockdown of *KCNJ5* or *GABRG3* or any of other 6 genes significantly increased the directedness (Supplementary Fig. 2). Seventeen gene knockdowns significantly affected the migration speed—*KCNA3*, *KCNA1* and seven other genes reduced the migration speed, while *CLIC3*, *AQP3* and six other genes increased the speed. The one exception is *ANO1*; after knockdown, both migration speed and directedness increased significantly (Supplementary Fig. 2). In a few cases, there appeared to be distinctively separate roles for the same category of genes in regulation of speed and directedness. Knockdown of ligand-gated Cl^−^ channels—*GABRG3*, *GABRQ* decreased the directedness without affecting migration speed, while the other family members *GABRR2*, *GLRA1* and *GLRA2* decreased the speed without significantly affecting the directedness (Supplementary Fig. 2). Voltage-gated K^+^ channels also showed similar separately regulated speed and directedness—*KCNA7*, *KCNAB1*, *KCNAB2* reduced directedness, while *KCNA1*, *KCNA3* decreased speed (Supplementary Fig. 2).

We performed a *z* score analysis which allows differentiation of more significantly different values from large samples (Fig. 1e). We set the cutoff value as a *z* score >0.495 or <−0.7, according to the upper and lower 2.5% of the distribution of the data, and this identified 18 genes. Knocking down nine candidates increased directedness, and knockdown of nine decreased directedness (Table 1). Knockdown of K^+^, Ca^2+^, Cl^−^ and non-selective cation channels showed significant decrease or increase in galvanotaxis. The 18 genes identified include five K^+^ channels (*KCNJ15*, *KCNJ5*, *KCNA7*, *KCNAB1* and *KCNAB2*), three that encode γ-subunits of voltage-gated Ca^2+^ channels (*CACNGs*—*CACNG3*, *CACNG5* and *CACNG8*), two *CLC* Cl^−^ channels, Ca^2+^-activated Cl^−^ channel (*ANO1*), two ligand-gated Cl^−^ channels (*GABRG3* and *GABRQ*), two purinergic receptors (*P2RX1* and *P2RX5*), water channel (*MIP*) and two other genes (*ENSA* and *TNFAIP1*).

### *KCNJ15* specifically mediated the field sensing

**Figure 2:**
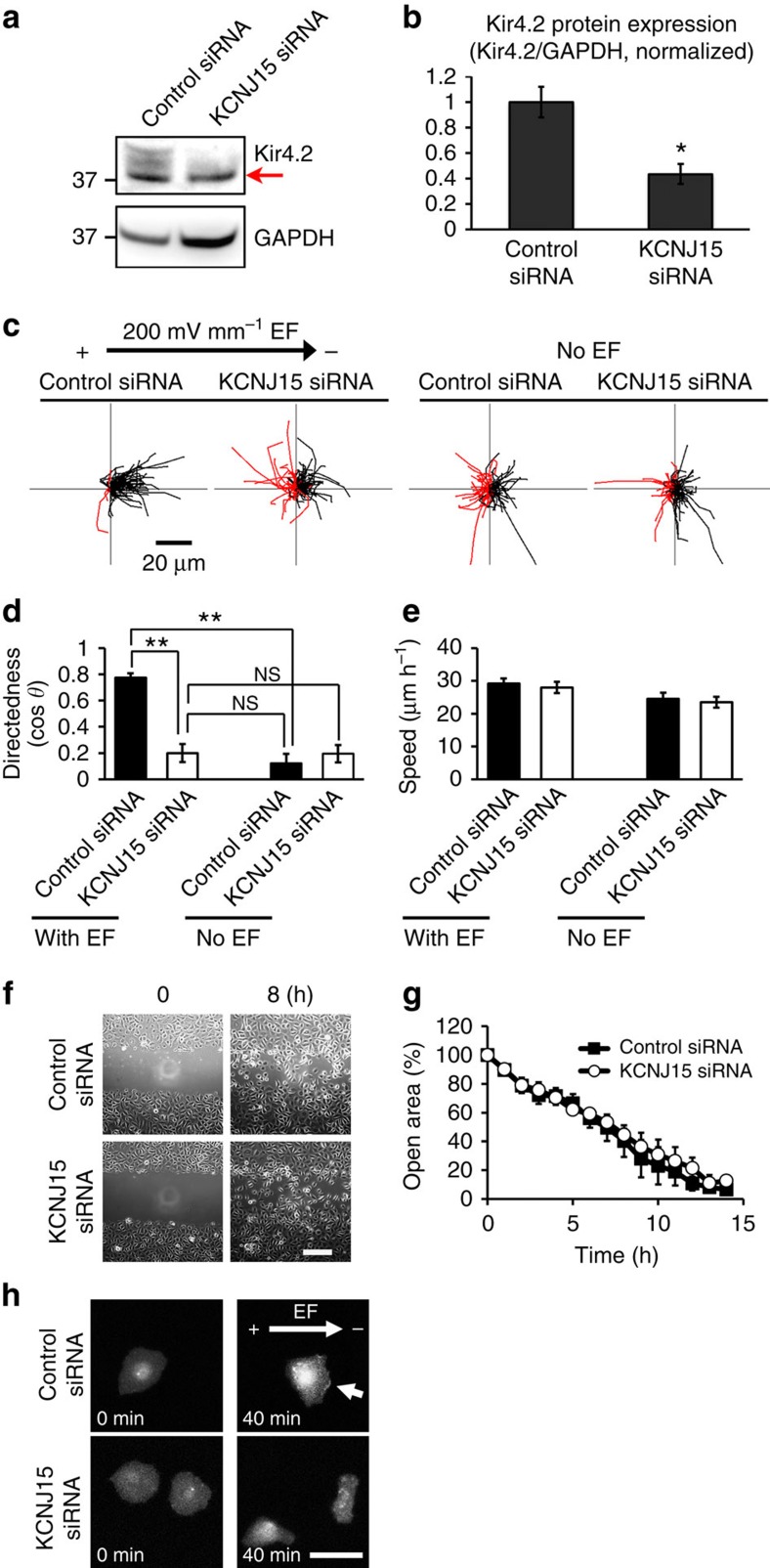
*KCNJ15* knockdown specifically abolished galvanotaxis. (**a**,**b**) Efficient knockdown of *KCNJ15* shown with Western blotting, red arrow pointing to a non-specific band. Kir4.2/GAPDH ratio is used to quantify the protein level. *n*=3. (**c**,**d**) Migration trajectories and quantification of directional migration (directedness values (cos *θ*)) demonstrated that *KCNJ15* knockdown abolished galvanotaxis and cells completely lost migration direction in an EF. Black and red lines indicate trajectories of cells migrated toward cathode and anode side, respectively. *n*=100 cells for each group, confirmed in two other replicates. (**e**) *KCNJ15* knockdown did not affect cell migration speed whether in an EF or not (compare the trajectories in c). There are no statistically significance between each group. *n*=100 cells for each group, confirmed in two other replicates. (**f**,**g**) Directional cell migration in scratch assay were identical between *KCNJ15* knockdown and scrambled RNAi treatment. Wound closure is represented as % of open area. When the error bars are not seen, the bars are smaller than the symbols. Wound was made using a pipette tip. There are no statistically significance between two groups. *n*=3. Scale bar in **f**, 200 μm. (**h**) *KCNJ15* knockdown abolished cathodal distribution of Akt-PH-EGFP, a reporter for PIP_3_ localization. hTCEpi cells were transfected with siRNA and pcDNA3-Akt-PH-EGFP plasmid DNA. Fluorescence of Akt-PH-EGFP was recorded by fluorescence microscope. Arrow indicates PIP_3_ accumulation in cathode-facing side of control cells. Scale bar, 50 μm. Cells were transfected with siRNA against *KCNJ15* or control oligo, and incubated for 48 h. EF=200 mV mm^−1^. Statistical analyses were performed by Student’s *t*-test. Data represented as mean±s.e.m. *P<0.05; **P<0.01. NS, not significant.

To minimize possible interference of decreased speed on quantification of directedness, we grouped genes according to the effects on migration speed and directedness after knockdown. We chose to focus on genes that after knockdown showed significantly decreased directedness without significant effect on migration speed (rose-coloured part in Supplementary Fig. 2). *KCNJ15* stood out; knockdown of *KCNJ15*, a gene encoding inwardly rectifying K^+^ channel Kir4.2, completely inhibited galvanotaxis while maintaining the same migration speed as non-target RNAi control (Table 1; Fig. 2c–e; Supplementary Video 1). Because this gene knockdown showed the most significant inhibition of directedness without affecting migration speed, we chose *KCNJ15* for further study. Knockdown efficiency was confirmed by real-time quantitative PCR (qPCR) and western blot for mRNA and protein, respectively. Transfection of siRNA against *KCNJ15* successfully reduced mRNA expression level by 80% (Supplementary Fig. 3a) and Kir4.2 protein level by 60% (Fig. 2a,b). Inwardly rectifying K^+^ channels, including *KCNJ15*/Kir4.2, are known to be important for the maintenance of resting membrane potential in various cells. We therefore measured the resting membrane potential of *KCNJ15* knocked down cells. Resting membrane potential of *KCNJ15* knocked down cells was significantly less negative (−38.98±0.66 mV; mean±s.e.m.) than that of control cells (−52.14±0.78 mV; Supplementary Fig. 4). To test whether other inward rectifying K^+^ channels may also participate in EF sensing, we tested *KCNJ10*/Kir4.1, which is also expressed in mouse corneal epithelial cells^20^. In the hTCEpi cells tested here, Kir4.1 appeared to localize exclusively in the perineuclear region (Supplementary Fig. 11, see below for details). Effective knocking down of *KCNJ10* had significantly less effect on the membrane potential (−48.57±1.04 mV from −52.14±0.78 mV) than knocking down of *KCNJ15* (Supplementary Figs 3b and 4), and also on galvanotaxis (cos *θ*=0.69±0.09 from 0.64±0.001) than knocking down of *KCNJ15* (cos *θ*=0.12±0.11 from 0.64±0.001; Supplementary Table 1). *KCNJ10*/Kir4.1 perhaps plays a lesser role in both galvanotaxis and resting membrane potential maintenance than *KCNJ15*/Kir4.2 in the hTCEpi cells.

**Figure 3:**
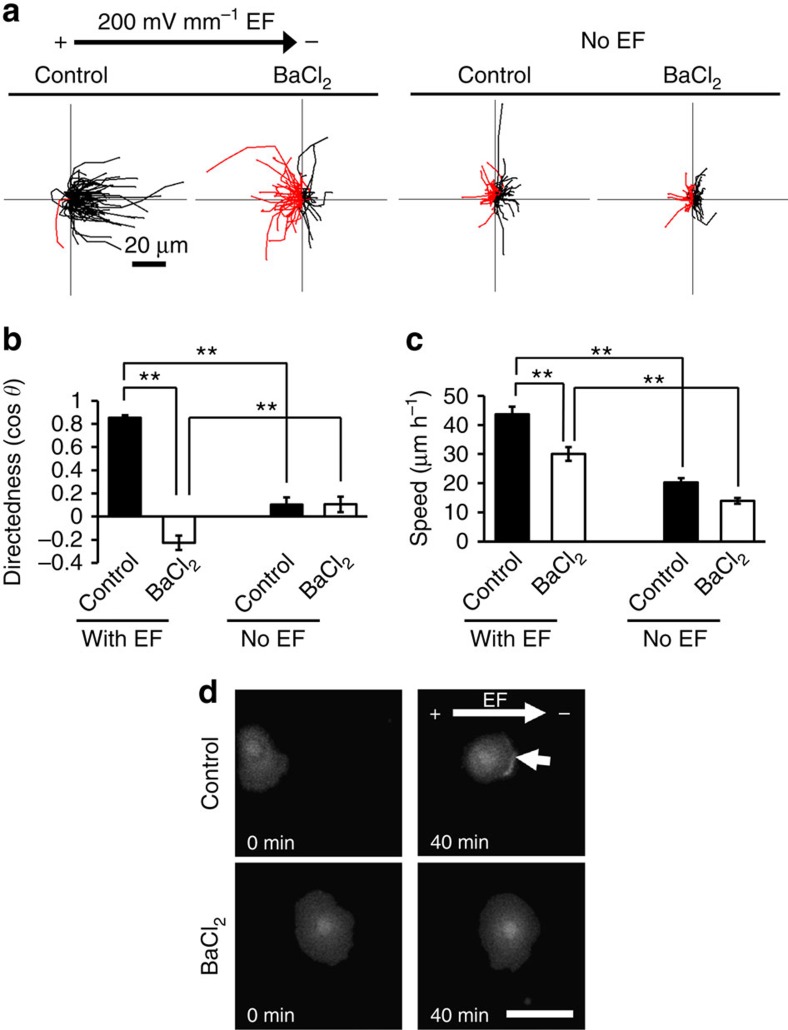
Barium chloride treatment abolished galvanotaxis. (**a**) Cells treated with BaCl_2_ lost galvanotaxis. Black and red lines indicate trajectories of cells migrated toward cathode and anode side, respectively. *n*=100 cells for each group, confirmed in two other replicates. (**b**) Directedness values (cos *θ*) confirm loss of directedness. *n*=100 cells for each group, confirmed in 2–3 other replicates. (**c**) BaCl_2_ treatment significantly inhibited migration speed. *n*=100 cells for each group, confirmed in 2–3 other replicates. (**d**) BaCl_2_ treatment prevented asymmetric accumulation of PIP_3_ to the leading edge. hTCEpi cells were transfected with pcDNA3-Akt-PH-EGFP plasmid DNA. Fluorescence of Akt-PH-EGFP was recorded by fluorescence microscope. Arrow indicates PIP_3_ accumulation in cathode-facing side of control cells. Scale bar, 50 μm. BaCl_2_ was used at 500 μM. EF=200 mV mm^−1^. Statistical analysis was performed by the Student’s *t*-test. Data represented as mean±s.e.m. **P<0.01.

To test the role of Kir4.2 with acute pharmacological treatment, we used Ba^2+^, a broad-range blocker for Kir channels. Ba^2+^ blocks inwardly rectifying K^+^ channels. Fifteen Kir channel-encoding genes (KCNJ1-6 and 8–16) have been identified in the human genome^21^, and Ba^2+^ inhibits them all. Ba^2+^ impaired galvanotaxis in a dose-dependent manner. Addition of BaCl_2_ (100 or 500 μM) caused complete loss of galvanotaxis of the cells with directedness values returning to around 0, and significantly decreased migration speed (Fig. 3 and Supplementary Video 2 for 500 μM BaCl_2_, Supplementary Fig. 5 for 100 μM BaCl_2_). Ba^2+^ inhibits Kir channels but not other types of K^+^ channels, such as voltage-gated K^+^ channels and Ca^2+^-activated K^+^ channels, at the concentration lower than millimolar order^22^.

We then investigated the specificity of *KCNJ15* in EF sensing. Cells after *KCNJ15* knockdown lost directedness in an EF, but maintained the same migration speed as non-target siRNA control cells or cells without an EF. The role for *KCNJ15* therefore appeared to be specific for directional sensing in an EF, not a general inhibition of cell motility (Fig. 2c–e). Migration trajectories of *KCNJ15* knockdown cells are similar to those of no EF cells (both control oligo- and *KCNJ15* siRNA-transfected cells). Cell migration in a monolayer scratch assay was identical in *KCNJ15* knockdown and non-target RNAi control. *KCNJ15* knockdown did not have any effect on wound closure, suggesting that the responsiveness of the cells to the directional cues (including injury, free edge and contact inhibition release) in this model remained the same (Fig. 2f). Several *KCNJ* genes are reported to be expressed in mouse corneal epithelial cells^20^,^23^. Knockdown of other *KCNJ* genes except *KCNJ14* (encoding Kir2.4) had no effect on the directedness (Supplementary Table 1). The inhibitory effect of BaCl_2_ was most likely through inhibition of Kir4.2 (*KCNJ15*). These results indicate that *KCNJ15* knockdown specifically affected sensing of the field, not motility or directional migration in a monolayer scratch assay.

The effects of *KCNJ15* knockdown on galvanotaxis at different EF strengths show the inhibition was complete up to 500 mV mm^−1^. Non-target control siRNA-transfected cells started to respond at 30 mV mm^−1^, and reached the maximum level at around 100 mV mm^−1^. BaCl_2_-treated cells showed the same loss of directedness in higher EF strength (Supplementary Fig. 6).

### Knockdown of *KCNJ15* prevented PIP_3_ polarization

Next, we determined the distribution of PIP_3_, a cell polarization marker, in cells after *KCNJ15* knockdown or Kir channel inhibition. Cells undergoing directional migration, including galvanotaxis, recruit PIP_3_ to the leading edge^8^,^24^,^25^,^26^. We transfected hTCEpi cells with *KCNJ15* siRNA followed by an expression construct of pleckstrin-homology domain of Akt fused with enhanced green fluorescence protein (Akt-PH-EGFP), or transfected with an Akt-PH-EGFP construct and treated with BaCl_2_. Akt-PH-EGFP reports PIP_3_ localization. In an EF, Akt-PH-EGFP redistributed to the cathode-facing side of hTCEpi cells. Cathode-polarization of Akt-PH-EGFP however was not observed in *KCNJ15* knockdown cells and BaCl_2_-treated cells (Figs 2h and 3d; Supplementary Tables 2 and 3).

### *KCNJ15*/Kir4.2 is also required for anode galvanotaxis

**Figure 4:**
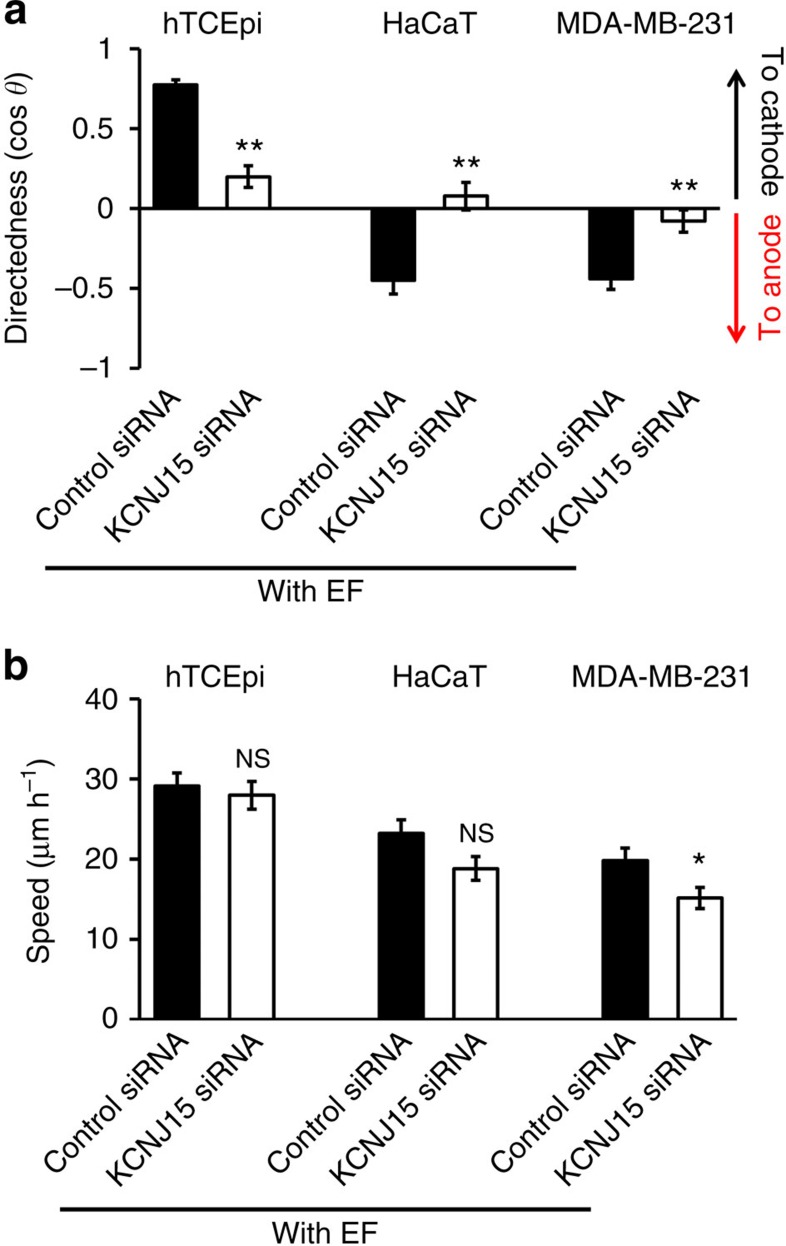
KCNJ15 knockdown abolished cathode as well as anode galvanotaxis. (**a**) hTCEpi cells migrate toward cathode, and HaCaT (human keratinocyte cells) and MDA-MB-231 (human breast cancer cells) cells migrate toward anode. Knockdown of KCNJ15 abolished directional migration in all three types of cells. At 48 h after transfection, cells were seeded onto galvanotaxis chamber. Positive directedness values indicate cathodal migration, whereas negative directedness value indicates anodal migration. (**b**) KCNJ15 knockdown did not have significant effects on migration speed. hTCEpi cells, HaCaT and MDA-MB-231 were transfected with siRNA against KCNJ15 or control oligo. Cell numbers analysed, 100 hTCEpi cells, 60 HaCaT cells and 80 MDA-MB-231 cells. Results confirmed in two separate experiments. Statistical analysis was performed by the Student’s *t*-test. Data represented as mean±s.e.m. *P<0.05. **P<0.01. EF=200 mV mm^−1^. NS, no significance.

In an EF, some types of cell migrate directionally to the anode, opposite to the direction of galvanotaxis of the corneal epithelial cells. To determine whether *KCNJ15* is required for anode galvanotaxis we transfected *KCNJ15* siRNA into two lines of anode-migrating cells. HaCaT cell (spontaneously immortalized human keratinocytes) and MDA-MB-231 cell (human breast adenocarcinoma line) migrated to the anode as shown by the negative directedness value (cos *θ*). Directional migration of both cell lines was lost after knockdown of *KCNJ15* (Fig. 4). KNCJ15/Kir4.2 thus is essential to both cathodal and anodal galvanotaxis. *KCNJ15* knockdown did not affect migration speed in HaCaT cells, as in hTCEpi cells. Knockdown of *KCNJ15* in MDA-MB-231 cells reduced migration speed as in mouse embryonic fibroblasts^27^. These observations may suggest that *KCNJ15* is specific in directional sensing in an EF; its involvement in regulating migration speed may be cell-type depend.

We also determined the distribution of PIP_3_ in anode-migrating HaCaT cells and MDA-MB-231 cells transiently transfected with Akt-PH-EGFP. No obvious polarized localization of Akt-PH-EGFP was observed in those anode-migrating cells (Supplementary Fig. 7).

### Kir4.2 coupled with polyamines to sense the EF

To elucidate the mechanisms of *KCNJ15*/Kir4.2 in sensing an EF, we examined the effects on galvanotaxis of pore blocking the Kir channels. Kir channels do not possess a canonical voltage sensing domain and have unique features unlike voltage-gated K^+^ channels^21^. Kir channels allow K^+^ more easily to flow into the cells than out of the cells. Intracellular polyamines regulate inward rectification activities of Kir channels. Polyamines are small organic compounds that have two or more primary amino groups, therefore carrying positive charges at regularly spaced intervals. In mammalian cells, spermidine (SPD), spermine (SPM) and putrescine (PUT) are three major polyamines. SPM and SPD, which have +4 and +3 charges, respectively, have enough size and charge to block Kir channels, whereas PUT does not. Highly positively charged polyamines bind to negatively charged residues, for example, glutamate and aspartate, located at the channel pore region. Polyamine depletion altered the inward rectifying property of Kir channels; that is, K^+^ flow reversed to outward rather than inward^28^,^29^,^30^,^31^.

**Figure 5:**
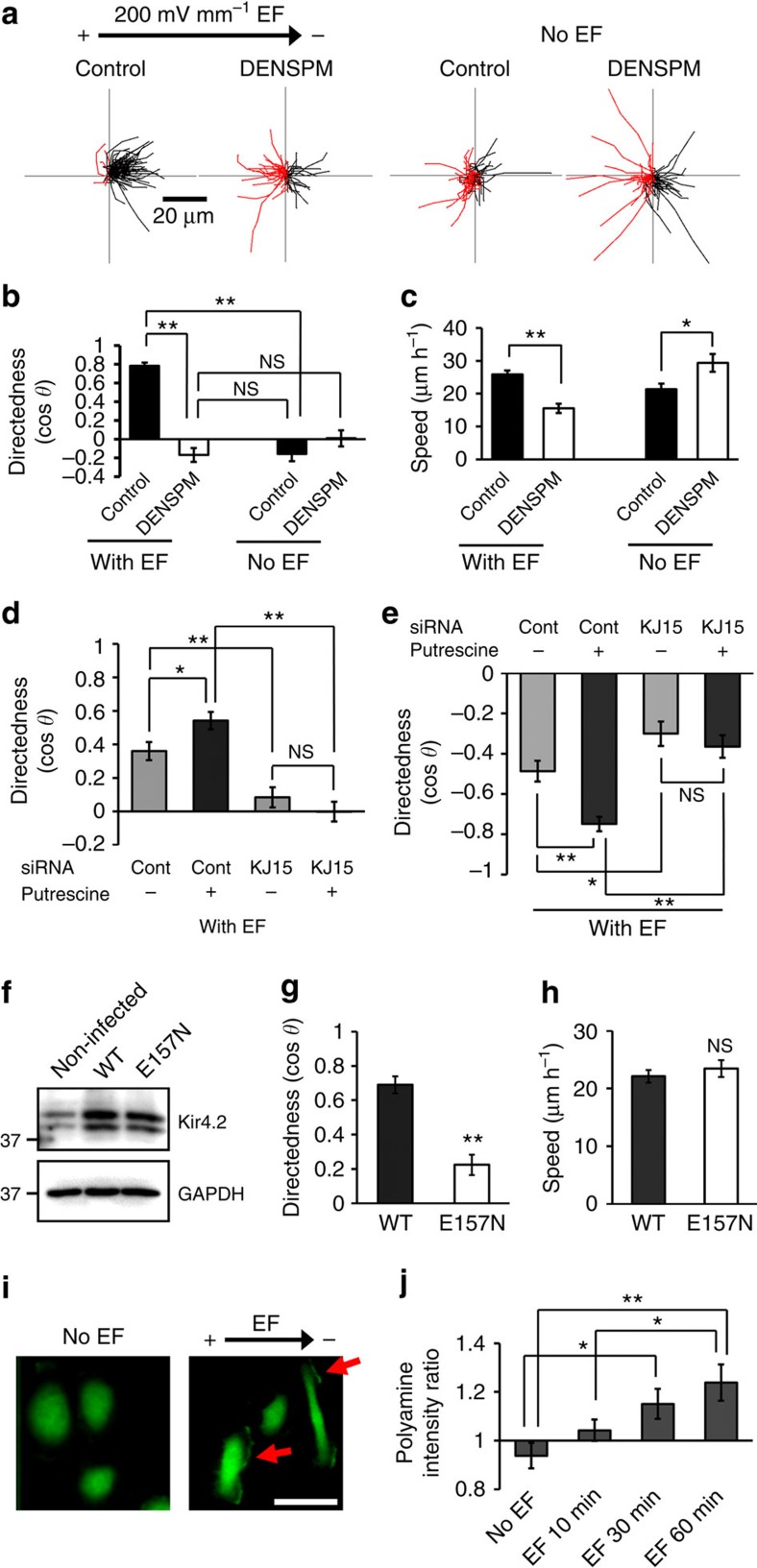
*KCNJ15* couples with polyamines to sense extracellular EFs. (**a**,**b**) Intracellular polyamines are required for cells to sense extracellular EFs. Depletion of polyamines with DENSPM abolished galvanotaxis. Migration trajectories of control cells and DENSPM treated cells with or without EF. Red lines indicate trajectories of cells that migrated toward anode side. hTCEpi cells were treated with 25 μM DENSPM for 2 days. DENSPM activates SPM/SPD catabolizing enzyme SAT/SSAT which catabolizes SPM/SPD to *N*^1^-acetyl SPM/SPD and reduces intracellular polyamine. (**c**) Depletion of polyamines affected cell migration speed. (**d**,**e**) Increased intracellular polyamines significantly enhanced galvanotaxis of U251 cells (**d**) or HaCaT cells (**e**). Knockdown of *KCNJ15* cancelled PUT-enhanced galvanotaxis. Cells were transfected with control oligo or *KCNJ15* siRNA, and treated with or without PUT (100 μM) for 2 days. (**f**) Lentivirus-mediated expression of WT and E157N Kir4.2 proteins. hTCEpi cells transduced with lentivirus. The Expression of WT and polyamine-binding defective mutant (E157N) Kir4.2 proteins was confirmed by western blotting. (**g**,**h**) Expression of polyamine-binding defective mutant of *KCNJ15* (E157N) significantly decreased directedness but had little effect on cell motility. hTCEpi cells were infected with recombinant lentivirus and incubated for 2 days. Directedness and speed were evaluated. (**i**,**j**) An applied EF-induced asymmetry of intracellular polyamines. Representative image of the polyamine staining (**i**). Scale bar in **i**, 50 μm. Intensities of polyamines staining in cathode-facing side were divided by those in anode-facing side (right side divided by left side in no EF cells) (**j**), Polyamine distribution in response to EF. hTCEpi cells were subjected to EF (200 mV mm^−1^) for 0, 10, 30 or 60 min. Intracellular polyamines were stained with anti-polyamine antibody. Arrows in **i** indicate polyamine accumulation in cathode-facing side of hTCEpi cells. Statistics: **b** and **c**: *n*=100 cells for each group, confirmed in three independent experiments. **d**: *n*=50, **e**: *n*=120, **g** and **h**: *n*=120, **j**: *n*=16–23. All confirmed in two to three separate experiments. EF=200 mV mm^−1^. Statistical analyses were performed by Student’s *t*-test (**b**–**e**,**g**,**h**), or analysis of variance followed by Student’s *t*-test (**j**). Data represented as mean±s.e.m. *P<0.05. **P<0.01. NS, no significance.

To test the role of polyamines (SPM/SPD) in galvanotaxis, we depleted intracellular polyamines by treating cells with polyamine analogue *N*^1^, *N*^11^-diethylnorspermine (DENSPM). Incubating cells with DENSPM, a potent activator of polyamine-catabolizing enzyme SPM/SPD acetyltransferase (SAT/SSAT), reduces intracellular SPM/SPD by catalysing the transacetylation reaction. Treatment with DENSPM completely abolished galvanotaxis (Supplementary Video 3; Fig. 5a–c). Cells migrated in random directions, as in *KCNJ15* knockdown and blocker experiments. Migration trajectories of DENSPM treated cells showed random migration similar to RNAi and blocker experiments (Figs 2 and 3; Supplementary Fig. 5).

We then increased the intracellular concentration of polyamines by incubating HaCaT and U251 cells with PUT, which is an important precursor of SPM/SPD synthesis. Treatment with PUT increases intracellular SPM/SPD concentrations^27^. PUT treatment significantly enhanced directedness of both HaCaT and U251 cells (Fig. 5d,e). The stimulatory effect of PUT on galvanotaxis was abolished by knocking down of *KCNJ15* in both HaCaT cells (anode migrating) and U251 (cathode migrating) cells. Importantly, knockdown of *KCNJ15* completely diminished PUT-induced enhancement of galvanotaxis (Fig. 5d,e).

To determine the role of interaction between channel protein and polyamines in galvanotaxis, we expressed polyamine-binding defective *KCNJ15*. Kir channels function as a tetramer in the plasma membrane. The pore region of Kir channels has a negatively charged amino-acid residue (corresponding to E157 of human Kir4.2), which interacts with polyamines. Mutation of this residue increased outward current^32^. Coexpression of wild type (WT) and mutant increased outward current that was intermediate between WT and mutant homo-tetramer^32^. Substitution of Glu-157 with Asn (E157N) resulted in complete loss of the inward rectification property of Kir4.2 (ref. 27). The mutant channels, if expressed in the cells, would act as ‘dominant negative’. We produced recombinant lentivirus to express mutant *KCNJ15* (E157N) and infected hTCEpi cells. Expression of E157N in hTCEpi cells decreased directedness (cos *θ*) but had little effect on cell motility (Fig. 5f–h). We further determined if expression of E157N affects PIP_3_ polarization in hTCEpi cells. We infected recombinant lentivirus to express WT or E157N, then transfected the expression construct of Akt-PH-EGFP. In WT expressing cells, about 38% of cells showed cathodal polarization of PIP_3_ (Akt-PH-EGFP) within 30 min after EF application. On the contrary, in E157N-expressing cells, only ∼24% of cells showed cathodal polarization of PIP_3_, which was significantly lower than WT expressing cells (*P*<0.01; Supplementary Fig. 8). These observations suggest that the interaction between Kir4.2 protein with intracellular polyamines is required to sense extracellular EFs, and modulation of the inward rectification properties of Kir4.2 by polyamines might be an important factor for polarization during galvatanoxis.

### EF-induced asymmetrical distribution of polyamines

To test if EFs cause asymmetrical distribution of these highly positively charged molecules, we applied an EF to hTCEpi cells and fixed them after different times in the EF (10, 30, 60 min and no EF control). The cells were stained with anti-polyamine antibody. SPM and SPD staining was much higher at the cathode-facing side than that at the anode-facing side (Fig. 5i,j). We measured the intensity of polyamine staining at both sides (cathode and anode, or right and left in no EF control) and calculated the cathode/anode or right/left ratios. Polyamines were accumulated at the cathode-facing side in response to EF application, and accumulation was increased in a time-dependent manner. Intracellular polyamines also accumulated at the cathode-facing side in response to EF in anode-migrating HaCaT and MDA-MB-231 cells (Supplementary Fig. 9)

We then tested if EFs cause asymmetrical distribution of Kir4.2 protein in hTCEpi cells. We applied an EF to hTCEpi cells and the cells were fixed and stained with anti-Kir4.2 antibody. F actin was visualized by using Alexa555-conjugated phalloidin. Kir4.2 protein was localized at the intracellular region (mostly perinuclear region), and the membrane expanding region (many dispersed dots in those regions). After application of EF, Kir4.2 protein signal was still observed in both cathode-facing and anode-facing sides, and intracellular region without obvious polarization (Supplementary Fig. 10).

Corneal epithelial cells express Kir4.1, which is encoded by *KCNJ10* (ref. 20) and has 62% amino-acid identity to Kir4.2. Immunostaining showed a different subcellular distribution of Kir4.1 from that of Kir4.2. Kir4.1 proteins were mainly localized at the intracellular perinuclear region and not expressed in the membrane expansion region (Supplementary Fig. 11). In addition, knocking down of *KCNJ10* had little effect on the directedness (cos *θ*; Supplementary Table 1). Consistently, membrane potential of *KCNJ10* knocked down cells was similar to that of control cells (Supplementary Fig. 4). These observations suggest that the functional contribution of *KCNJ10*/Kir4.1 to maintenance of resting membrane potential and galvanotaxis is significantly smaller than that of *KCNJ15*/Kir4.2.

## Discussion

Important intracellular signalling mediators for galvanotaxis have been identified, for example, phosphatidylinositol-3-OH kinase γ (PI3Kγ), PTEN, Src, Rac, cAMP and cGMP (refs 26, 33, 34, 35, 36). The proximal sensor molecule(s) and/or sensing mechanism(s) remain elusive. Previous studies suggested some potential molecule ‘sensor(s)’, including epithelial sodium channel (ENaC) in keratinocytes, voltage-gated sodium channel in prostate cancer cells and potassium transporters in yeasts^37^,^38^,^39^,^40^,^41^,^42^. Voltage-gated sodium, potassium and calcium channels (Nav, Kv and Cav) possess a voltage sensing domain and respond to membrane potential change. However, these channel proteins can usually only respond to large potential change (10 mV or more across the cell membrane)^43^. This is one to several orders larger than the threshold fields that can induce marked electrotaxis (∼0.2–2 mV across a cell of 20 μm diameter in a field of 10–100 mV mm^−1^). No known molecules are able to detect such a small field, which only induces less than ∼1% change in membrane potential.

Potassium channels are implicated in migration in epithelial cells, neutrophils, astrocytes and fibroblasts, presumably through ion influx or efflux, which would generate a driving force for water flow at both leading and trailing edges through aquaporin water channels^27^,^44^,^45^,^46^,^47^,^48^. Kir4.2 is an inward rectifying K^+^ channel that co-localizes with α9β1 integrin and SAT/SSAT at the leading edge in glioma and Chinese hamster ovary cells. SAT/SSAT decreases local SPM/SPD concentration and allows K^+^ efflux locally to regulate cell migration^27^,^48^.

*KCNJ15* was one of a group of genes whose knockdown showed significantly decreased directedness but less effect on speed of cell migration (Supplementary Fig. 2, rose-coloured part). In addition to *KCNJ15*, three other K^+^ channel-encoding genes (*KCNA7*, *KCNAB1* and *KCNAB2*), one cation permeable channel gene (*P2RX5*), two GABA receptor subunit coding genes (*GABRG3* and *GABRQ*) and two other genes *ENSA* and *TNFAIP1* fell into this group. The product of *ENSA* may regulate the ATP-sensitive K^+^ (K_ATP_) channel. The majority of the gene targets appear to relate to K^+^ fluxes. In a recent elegant study using budding yeast (*Saccharomyces cerevisiae*), Haupt *et al.* demonstrated that K^+^ transporter Trk1p is a key molecule for EF-induced budding and polarization, which may be mediated by Cdc42. Using light-sensitive ion channels expressed in yeast, the site of polarization could be controlled using a focused laser beam^42^. Our results in human cells reported here and the identification of K^+^ transport in yeast polarization by Haupt *et al.* suggest a conserved role for K^+^ flux in integration of electrical regulation into much better understood biochemical pathways in cell polarity, for example, through cdc42. Further investigation on K^+^ in regulation of cell polarization and directional migration will likely provide significant insights. Some of those channels are known to be involved in membrane potential regulation. Indeed, knockdown of *KCNJ15* significantly depolarized the cells. These different channel targets offer a great basis to answer whether channels other than K^+^ channels share the same signalling transduction mechanism, and how they converge to result in directional polarization and migration in an EF.

In hTCEpi cells, inhibition of Kir4.2 by knockdown of *KCNJ15* or BaCl_2_ treatment, or depletion of intracellular SPM/SPD, abolished galvanotaxis without affecting motility. Cells establish front-rear polarity and maintain the polarity during migration^49^. Establishment of polarity requires the breaking of cell symmetry^50^. Many signalling molecules, such as small GTPases (Rho, Rac and Cdc42), atypical PKC (aPKC) and PIP_3_/PIP_2_ regulate and maintain front-rear polarity^51^,^52^,^53^,^54^. PIP_3_ accumulates at the front of *Dictyostelium* cells and neutrophils in establishment and maintenance of polarity in chemotaxis as well as in electrotaxis^26^,^36^,^55^,^56^,^57^. *KCNJ15* knockdown and BaCl_2_ treatment abolished PIP_3_ accumulation at the front (Figs 2h and 3d), and these cells did not migrate directionally (Figs 2c–e and 3a–c). Furthermore, the expression of polyamine-binding defective mutant (E157N) partially inhibited PIP_3_ polarization in the front of hTCEpi cells (Supplementary Fig. 8). These results suggest that *KCNJ15*/Kir4.2 and its interaction with polyamines are essential for sensing an EF.

In anode-migrating HaCaT and MDA-MB-231 cells, knocking down of *KCNJ15* or manipulation of intracellular polyamines also affected galvanotaxis significantly (Figs 4 and 5e). These observations suggest that *KCNJ15*/Kir4.2 and polyamines are also important for anodal galvanotaxis. Polarization of anode-migrating cells under EFs might be regulated by different mechanism(s). For example, our recent study shows that fish keratocyte and fragments derived from parental keratocyte use competing mechanisms to migrate in opposite directions in response to EFs (ref. 58). Poo and colleagues have reported that differences in cyclic AMP-dependent activity in a neuronal cell result in opposite turning of the growth cone in response to the same guidance cue (for example, Netrin-1)^59^,^60^. Xu *et al.* reported that inhibition of Gi with pertussis toxin caused HL-60 cells to polarize away from chemoattractants^61^. These observations suggest that presetting of the intracellular signalling networks may determine cell polarization direction so the cells may polarize in opposite directions to the same stimulus.

It has been suggested that intracellular charged small molecules migrate in a certain direction (positively charged molecules migrate to negative pole, and negatively charged molecules migrate to positive pole) when the cells are exposed to a physiological extracellular EF (ref. 62). Cooper *et al.*^63^ demonstrated that an applied EF redistributed injected dye molecules with positive charge in a crayfish nerve cord. The dye moved toward the negative pole in response to an extracellular EF, and could even migrate to the neighbour cells through gap junctions. Applied EFs induced intracellular polyamines to accumulate at the cathode-facing edge (Fig. 5i,j). On the other hand, localization of Kir4.2 protein was not affected by applied EFs (Supplementary Fig. 10). Asymmetrically redistributed polyamines bind to Kir4.2 to regulate K^+^ fluxes. Indeed, expression of mutated molecule with defective polyamine-binding site in KCNJ15-E157N significantly decreased the electrotaxis response (Fig. 5f–h), suggesting the necessity of Kir4.2-polyamine interaction in regulation of electrotaxis.

We propose here a two-molecule coupling model in galvanotaxis. Weak extracellular EFs redistribute positively charged polyamines, which then bind to Kir4.2 to regulate the K^+^ fluxes. Importantly, this two-molecule mediated sensing mechanism appears to be essential for both cathode- and anode-migrating cells (Fig. 4a). We speculate that Kir4.2 activity-induced local changes in membrane potential, osmolality and ionic environment may affect the well-established PI3K/Akt pathway, and eventually affect actin polymerization and membrane protrusion^64^,^65^. There may be direct interaction between Kir channel(s) and Akt, which is downstream of PIP_3_ (refs 66, 67; Supplementary Fig. 12).

In conclusion, we have developed an effective screening strategy to profile the galvanotaxis phenotype in large numbers of different types of cells from libraries, either through RNAi knockdown, mutation or other treatment. This approach identified a group of key channel genes and proteins that are critical for galvanotaxis. Interestingly, two categories of channel are found—enhancers and inhibitors. Among them, *KCNJ15* is an essential gene for sensing extracellular EFs in human epithelial cells. Our data suggest a novel two-molecule model by which Kir4.2 interacts with intracellular polyamines in sensing weak extracellular EFs in galvanotaxis.

## Methods

### Materials

EpiLife culture medium with Ca^2+^ (60 μM), EpiLife defined growth supplement, DMEM, foetal bovine serum (FBS), non-essential amino-acids solution ( × 100), penicillin/streptomycin, BlockiT red fluorescent oligo and Lipofectamine 2000 reagent were purchased from Life Technologies Inc. (Carlsbad, CA, USA). X-tremeGENE HD DNA transfection reagent was purchased from Roche Applied Science (Penzberg, Upper Bavaria, Germany). On-target plus siRNA human ion channel siRNA library was purchased from Thermo Fisher Scientific (Waltham, MA, USA). pcDNA3-Akt-PH-EGFP (addgene plasmid 18836) was described in ref. 68 and purchased from addgene (Cambridge, MA, USA). Anti-KCNJ15 polyclonal antibody (Cat. No. 15988-1-Ab, dilution 1:500) was purchased from Proteintech (Chicago, IL, USA). Anti-Kir4.1 polyclonal (Cat. No. APC-035, dilution 1:50) and anti-Kir4.2 polyclonal (Cat. No. APC-058, dilution 1:50) antibodies were purchased from Alomone Labs (Jerusalem, Israel). Anti-SPM polyclonal antibody (Cat. No. ab26975, dilution 1:50–1:100) was purchased from Abcam (Cambridge, MA, USA). Anti-GAPDH polyclonal antibody (Cat. No. sc-25778, dilution 1:1,000) was purchased from Santa Cruz Biotechnology (Santa Cruz, CA, USA). FNC Coating Mix was purchased from Athena ES (Baltimore, MD, USA). RIPA buffer was purchased from EMD Millipore (Billerica, MA, USA). RNeasy mini kit was purchased from QIAGEN (Venlo, Netherlands).

### Cell culture

Telomerase immortalized hTCEpi cells were generously provided by Dr James Jester (University of California, Irvine). HaCaT cells, derived from spontaneously immortalized human keratinocyte, were generously provided by Dr Fu-Tong Liu (University of California Davis). U251 cells, derived from human glioblastoma multiforme, were generously provided by Dr Garret Yount (California Pacific Medical Center Research Institute). MDA-MB-231 cells, derived from human breast adenocarcinoma, were generously provided by Dr Yoshikazu Takada (UC Davis). hTCEpi cells were grown in EpiLife with 60 μM Ca^2+^ supplemented with EpiLife defined growth supplement and penicillin/streptomycin at 37 °C with air containing 5% CO_2_. We used hTCEpi cells between passage number 50 and 70. HaCaT cells and U251 were grown in DMEM supplemented with 10% FBS and penicillin/streptomycin at 37 °C with air containing 5% CO_2_. MDA-MB-231 cells were grown in DMEM supplemented with 10% FBS, 1 × non-essential amino acids and penicillin/streptomycin at 37 °C with air containing 5% CO_2_.

### EF-induced migration

Direct current was applied through agar-salt bridges connecting with silver/silver chloride electrodes in Steinberg’s solution (consisting 58 mM NaCl, 0.67 mM KCl and 0.44 mM Ca(NO_3_)_2_, 1.3 mM MgSO_4_ and 4.6 mM Tris base, pH 7.4) to pooled medium on either side of the galvanotaxis chamber. Cells were exposed to 0–500 mV mm^−1^ direct current EF for 30 min. We normally applied 200 mV mm^−1^ EF unless otherwise noted. Cell migration was observed with a Carl Zeiss Axiovert 40 CFL inverted microscope with Simple PCI program (Hamamatsu corp., Sewickley, PA, USA) or Carl Zeiss Observer Z1 inverted microscope with MetaMorph NX program (Molecular Devices, Sunnyvale, CA, USA), and serial time-lapse images were captured. Cell migration was analysed to determine directedness (cos *θ*) and track speed by using ImageJ software (NIH, Bethesda, MA, USA) with MTrackJ and Chemotaxis tool plugins^69^. Briefly, trajectories of cells were pooled to make composite graphs. The directedness of migration was assessed as cos *θ*, where *θ* is the angle between the EF vector and a straight line connecting start and end positions of a cell. A cell moving directly to cathode would have a directedness of 1; a cell moving directly to the anode would have a directedness of –1. A value close to 0 represents random cell movement. Speed is the total length travelled by the cells divided by time.

### Screening of RNAi library

We used on-target plus siRNA human ion channel siRNA library. hTCEpi cells were seeded at the density of 5 × 10^4^ cells per well in 12-well plate 1 day before transfection. We transfected cells with individual siRNA separately using Lipofectamine 2000 reagent according to the manufacturer’s protocol. At 48 h after transfection, cells were trypsinized and seeded onto electrotaxis chamber pre-coated with FNC Coating Mix, as shown in Fig. 1. EF (200 mV mm^−1^) was applied for 30 min, cell migration was recorded, and directedness and track speed was determined as described above. *z* score was calculated using the formula *z*=(X−μ)/s.d. where X is the sample value of directedness (cos *θ*), μ is the mean of the whole population and s.d. is the standard deviation of the whole population. We picked genes with *z* scope >0.495 or <−0.7 to suggest genes that after knockdown resulted in directedness value or migration speed in the 2.5% upper or lower distribution.

### Wound scratch assay

hTCEpi cells were seeded at a density of 1.5 × 10^5^ cells per well in 12-well plate 1 day before transfection. The cells were transfected with siRNA as described above. At 48 h after transfection a scratch wound was made using a pipette tip. Wound closure was observed with a microscope for 14 h.

### Isolation of RNA and real-time qPCR

Total RNA was isolated by using the RNeasy mini kit according to the manufacturer’s protocol. First strand cDNA was synthesized by using SuperScript III 1st strand cDNA synthesis kit (Life Technologies). Real-time qPCR was performed by using SsoAdvanced SYBR green master mix (BIORAD). We used glyceraldehyde-3-phosphate dehydrogenase (GAPDH) as an internal standard. Primer sequences are as follow;

KCNJ15 set1 sense, 5′-TGAGATCTTCATCACCGGAAC-3′

KCNJ15 set1 antisense 5′-TTGGCTACCTGAATCACCAAG-3′

KCNJ15 set2 sense, 5′-AGTCATCACCAAGCAGAATGG-3′

KCNJ15 set2 antisense 5′-TTGGCTACCTGAATCACCAAG-3′

KCNJ10 set1 sense 5′-AACCAAGGAAGGGGAGAACATC-3′

KCNJ10 set1 antisense 5′-GGGTAGAATAAGGAAGGGGCTG-3′

KCNJ10 set2 sense 5′-GTGGTGTGGTATCTGGTAGCTG-3′

KCNJ10 set2 antisense 5′-ATTCAAGGGAGAAGAGGAAGGC-3′

GAPDH sense, 5′-GAAGGTCAAGGTCGGAGTC-3′

GAPDH antisense, 5′-CAAGATGGTGATGGGATTTC-3′

### Western blotting

hTCEpi cells transfected with siRNA were lysed with RIPA buffer at 48 h after transfection. Equal amount of proteins (25 μg) were separated by SDS-PAGE (10% gel), transferred onto PVDF membrane. Protein bands were visualized by enhanced chemiluminescence method. Uncropped western blots appear in Supplementary Fig. 13.

### Imaging of Akt-PH-EGFP

hTCEpi cells were seeded at a density of 1.2 × 10^5^ cells per well in a 12-well plate 1 day before transfection. The cells were transfected with 500 ng per well of pcDNA3-Akt-PH-EGFP plasmid using X-tremeGENE HD DNA transfection reagent according to the manufacturer’s protocol. At 18–24 h after transfection, the cells were trypsinized and seeded onto electrotaxis chamber pre-coated with FNC coating mix, as in EF-induced migration experiments. The cells were exposed to 200 mV mm^−1^ EF. Serial time-lapse EGFP fluorescence and phase contrast images were captured.

### Electrophysiology

siRNA-transfected cells were visualized by an inverted microscope (Olympus IX71) placed in a Faraday cage on an anti-vibration table. Electrical signals were recorded with intracellular amplifiers (Axopatch 200B, Axon Instruments) after analogue-digital conversion (Digidata 1440A, Molecular Devices) and analysed using pClamp 10 software (Molecular Devices). Membrane potentials were recorded in current-clamp mode with sharp borosilicate microelectrodes filled with 3 M KCl having a tip resistance of 30–40 MΩ. Using this technique the cytosol was not dialyzed. Experiments were done at 22 °C in EpiLife medium.

### Polyamine staining

hTCEpi cells were trypsinized and seeded onto electrotaxis chamber pre-coated with FNC coating mix. The cells were exposed to 200 mV mm^−1^ EF for 0, 10, 30 and 60 min. The cells were quickly fixed with 4% paraformaldehyde, stained with anti-SPM antibody and observed with fluorescence microscopy. Intensities of SPM/SPD were measured using ImageJ software with Colour functions plugin.

### Lentivirus production and transduction

pSIN-Luc-Ub-Em and pSIN-CSGWdlNotI lentivirus gene transfer vector was kindly provided by Dr Yasuhiro Ikeda (Mayo Clinic)^70^. Sequence-verified cDNA of human KCNJ15 was purchased from GE healthcare (Little Chalfont, UK). We amplified full-length KCNJ15 cDNA by using KCNJ15 forward and KCNJ15 reverse primers, digested with *Bam*HI and *Not*I, and once inserted into same site of pcDNA3.1 vector. Site-directed mutagenesis was performed by self-ligation of inverse PCR product (SLIP) method by using pcDNA-KCNJ15 WT as a template and SLIP forward and SLIP reverse primers.

Primer sequences are as follow;

KCNJ15 forward, 5′-AAAGGATCCCTGGCAATGGATGCCATTCACATCGGC-3′

KCNJ15 reverse, 5′-AAAGCGGCCGCTCAGACATTGCTCTGTTGTAATAAAAGTG-3′

SLIP forward, 5′-AACATCTTCATCACCGGAACCTTCC-3′

SLIP reverse, 5′-AATCAAGGTCGTGATGACCAACTG-3′

Single and double underlines indicate restriction sites (*Bam*HI and *Not*I) and mutagenesis site, respectively. pSIN-Luc-Ub-Em or pSIN-CSGWdlNotI vector was digested with *Bam*HI and *Not*I. cDNA encoding WT or E157N KCNJ15 were recovered from pcDNA-KCNJ15 WT or E157N by *Bam*HI and *Not*I digestion, and inserted into pSIN-Luc-Ub-Em or pSIN-CSGWdlNotI vector digested with *Bam*HI and *Not*I. We confirmed the sequence of each one by DNA sequencing. For lentivirus packaging, we transfected pSin-KCNJ15 WT or pSIN-KCNJ15 E157N vector into 293T cells together with the packaging plasmid and the envelope plasmid, and the supernatant fraction containing lentivirus particles was collected at 48 h after transfection. hTCEpi cells were infected with lentivirus. At 48 h after infection, cell migration was evaluated.

### Statistics

All data are represented as means±s.e.m. analysis of variance and the Student’s *t*-test were used for statistical analysis as appropriate and a *P* value <0.05 was considered as statistically significant.

## Additional information

**How to cite this article:** Nakajima, K. *et al.* *KCNJ15*/Kir4.2 couples with polyamines to sense weak extracellular electric fields in galvanotaxis. *Nat. Commun.* 6:8532 doi: 10.1038/ncomms9532 (2015).

## Supplementary Material

Supplementary Information Supplementary Figures 1-13 and Supplementary Tables 1-3

Supplementary Movie 1 Knockdown of KCNJ15 abolished galvanotaxis. hTCEpi cells were transfected with siRNA against KCNJ15 or control oligo. Transfected cells were seeded onto galvanotaxis chamber at 48 h after transfection. Cell migration was recorded by microscope. Bar, 100 μm.

Supplementary Movie 2 Barium chloride abolished galvanotaxis. hTCEpi cells were treated with BaCl2 (500 μM). Cell migration was recorded by microscope. Bar, 100 μm.

Supplementary Movie 3 Exhaustion of polyamine with N1, N11-diethylnorspermine (DENSPM)

## Figures and Tables

**Table 1:** Genes which after knocking down caused impaired or enhanced galvanotaxis.

**Effect**	**Gene**	**Directedness (cos** ***θ*)**
Control		0.635±0.000819
Impaired	*ENSA*	0.269±0.108
	*GABRG3*	0.201±0.0707
	*GABRQ*	0.251±0.0753
	*KCNA7*	0.196±0.0714
	*KCNAB1*	0.276±0.0741
	*KCNAB2*	0.241±0.102
	*KCNJ15*	0.115±0.107
	*P2RX5*	0.140±0.0967
	*TNFAIP1*	0.281±0.113
Enhanced	*CACNG3*	0.879±0.0274
	*CACNG5*	0.875±0.0349
	*CACNG8*	0.900±0.0526
	*CLCN1*	0.876±0.0290
	*CLCN5*	0.915±0.0173
	*P2RX1*	0.921±0.0140
	*ANO1*	0.895±0.0336
	*MIP*	0.891±0.0179
	*KCNJ5*	0.873±0.0316

Directedness (cos *θ*) of candidate genes is represented as mean±s.e.m. (selected from Fig. 1d). EF=200 mV mm−1.
