# Supplementary material for: KCNJ15/Kir4.2 couples with polyamines to sense weak extracellular electric fields in galvanotaxis
Source: Nat Commun. 2015 Oct 9;6:8532. doi: 10.1038/ncomms9532 (PMC4603535; doi:10.1038/ncomms9532)
Supplement: Supplementary Information — Supplementary Figures 1-13 and Supplementary Tables 1-3 [file ncomms9532-s1.pdf]

Supplementary Figure 1

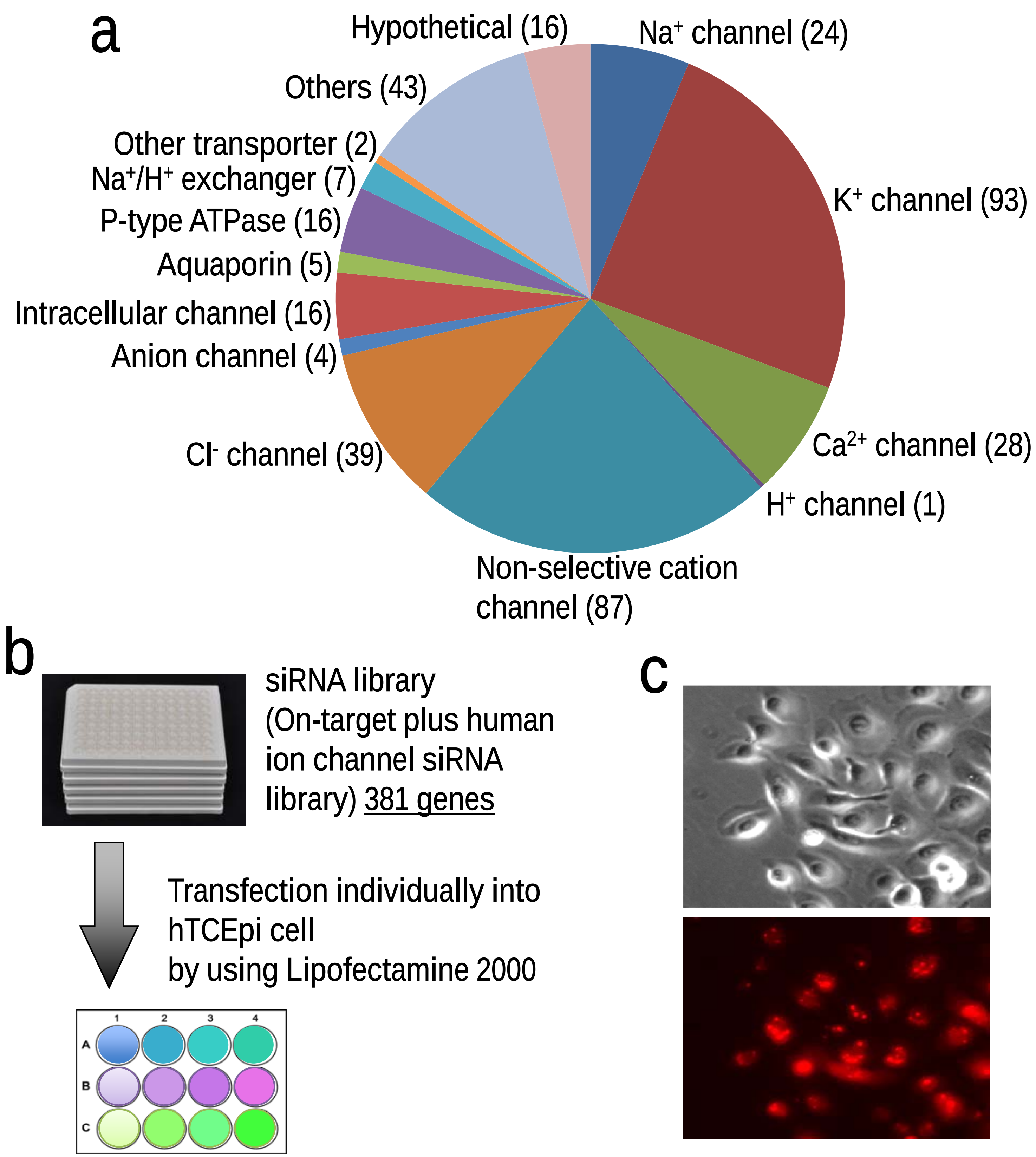

Supplementary Figure 1. RNAi targeting ion channels and transporters.

(a) On-Target plus human ion channel siRNA library.

(b) Knockdown of multiple genes at the same time in separate wells. Cells were seeded into 12 well plate at the density of 5×10^4 cells/well, and transfected with siRNA using Lipofectamine 2000 reagent.

(c) Fluorescence image (fluorescent-labeled oligo) demonstrates high transfection efficiency in hTCEpi cells.

Supplementary Figure 2

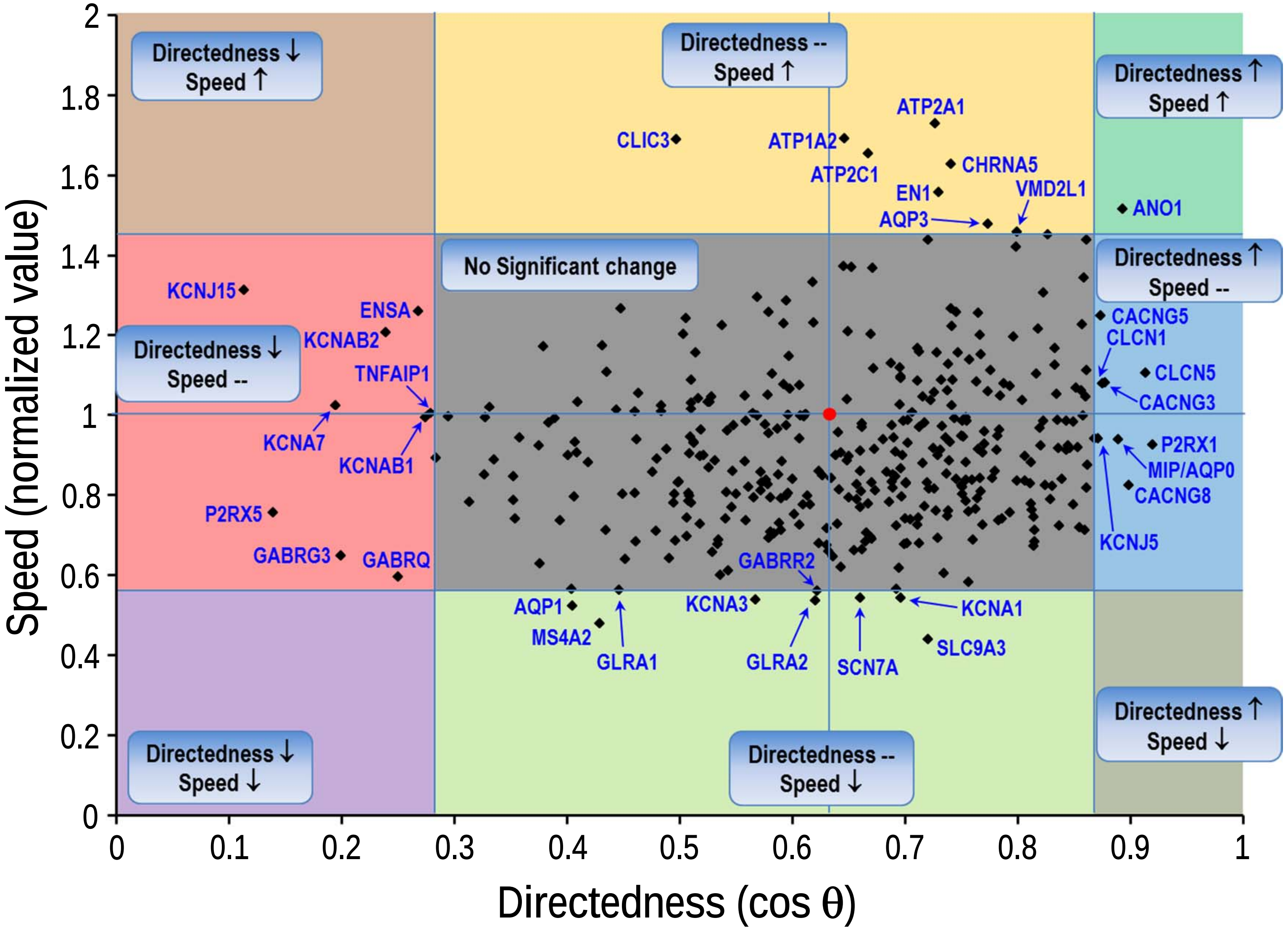

Supplementary Figure 2. Distinct effects on migration rate and directionality after knockdown of genes.

Directedness (X axis) plotted against migration speed (Y axis). Each dot represents migration rate and directedness after knockdown of individual gene. The upper and lower cut off line at 2.5% of the directedness value and migration speed are separately plotted, and grouped as 9 groups. The effects of knockdown on directedness and migration speed are not the same. Four major groups are increased or decreased directedness, and increased or decreased speed. Genes with significant effects are labelled. For example, KCNJ15 knockdown reduced directedness without affecting speed significantly, and CLIC3 knockdown increased speed without affecting directedness significantly. Only one exception is ANO1 knockdown, which enhanced both directedness and migration speed. Red circle on the center is control (cos θ=0.635, normalized speed=1). Significant level is set at 0.025.

Supplementary Figure 3

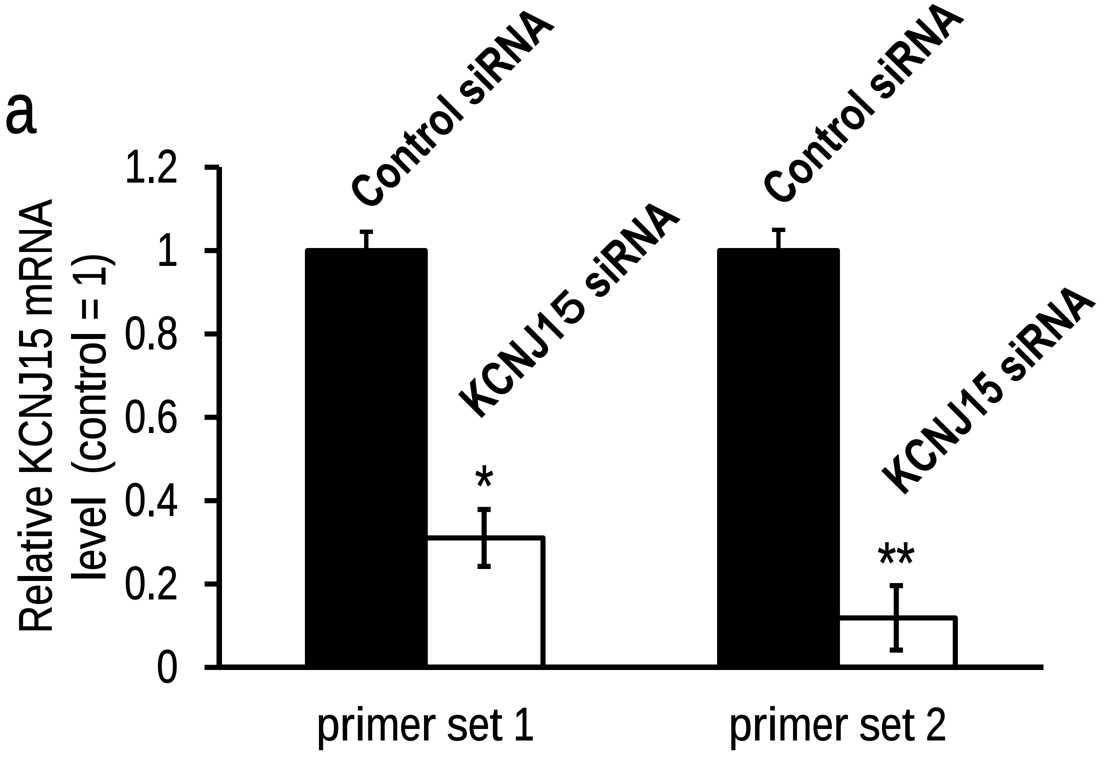

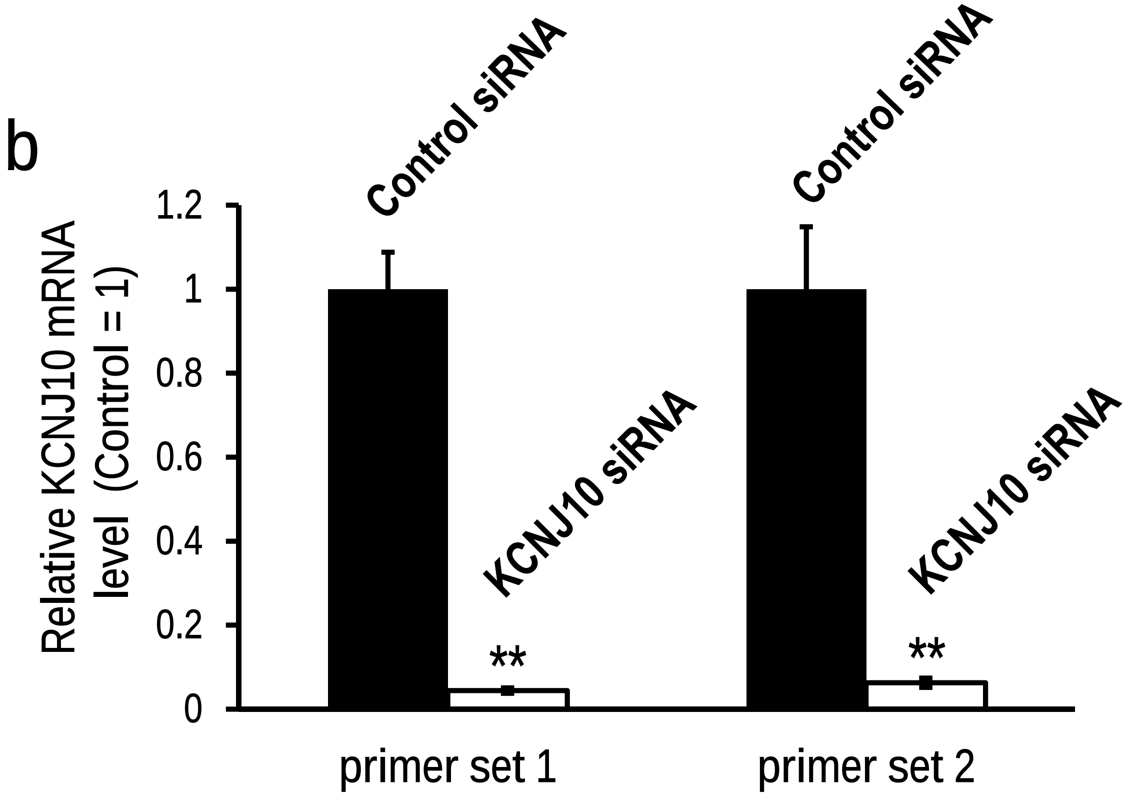

Supplementary Figure 3. Effective knockdown of KCNJ15 confirmed by realtime qPCR.

(a) Quantification of KCNJ15 mRNA levels in control and KCNJ15 knockdown cells by realtime quantitative PCR. We used two independent primer sets (primer set 1 and 2) (see Methods). Both showed very efficient knockdown of KCNJ15.

(b) Quantification of KCNJ10 mRNA levels in control and KCNJ10 knockdown cells by realtime quantitative PCR. We used two independent primer sets.

n=3. Statistical analysis was performed by the Student's *t*-test.

Data represented as mean ± SEM. *, $p<0.05$. **, $p<0.01$.

Supplementary Figure 4

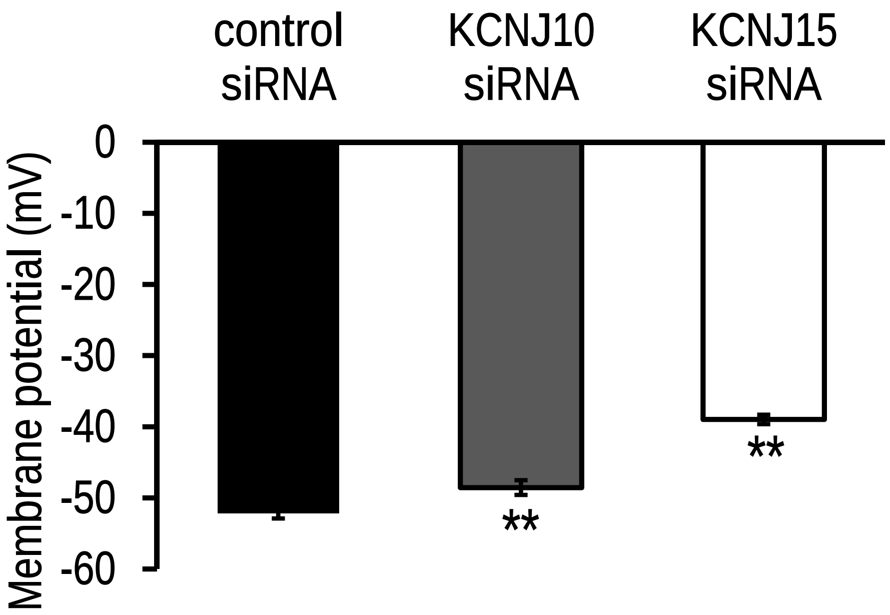

Supplementary Figure 4. Knocking down KCNJ15 had a significantly more effect on the resting membrane potential than knockdown of KCNJ10.
hTCEpi cells were transfected with siRNA against KCNJ10 or KCNJ15, or control siRNA for 2 days. Membrane potentials were recorded in current-clamp mode with sharp borosilicate microelectrodes filled with 3 M KCl having a tip resistance of 30–40 MΩ. *n*=141 for control group, *n*=66 for KCNJ10 knock down group, *n*=54 for KCNJ15 knock down group. Statistical analysis was performed by the Student's *t*-test. Data represented as mean ± SEM.
**, p<0.01.

Supplementary Figure 5

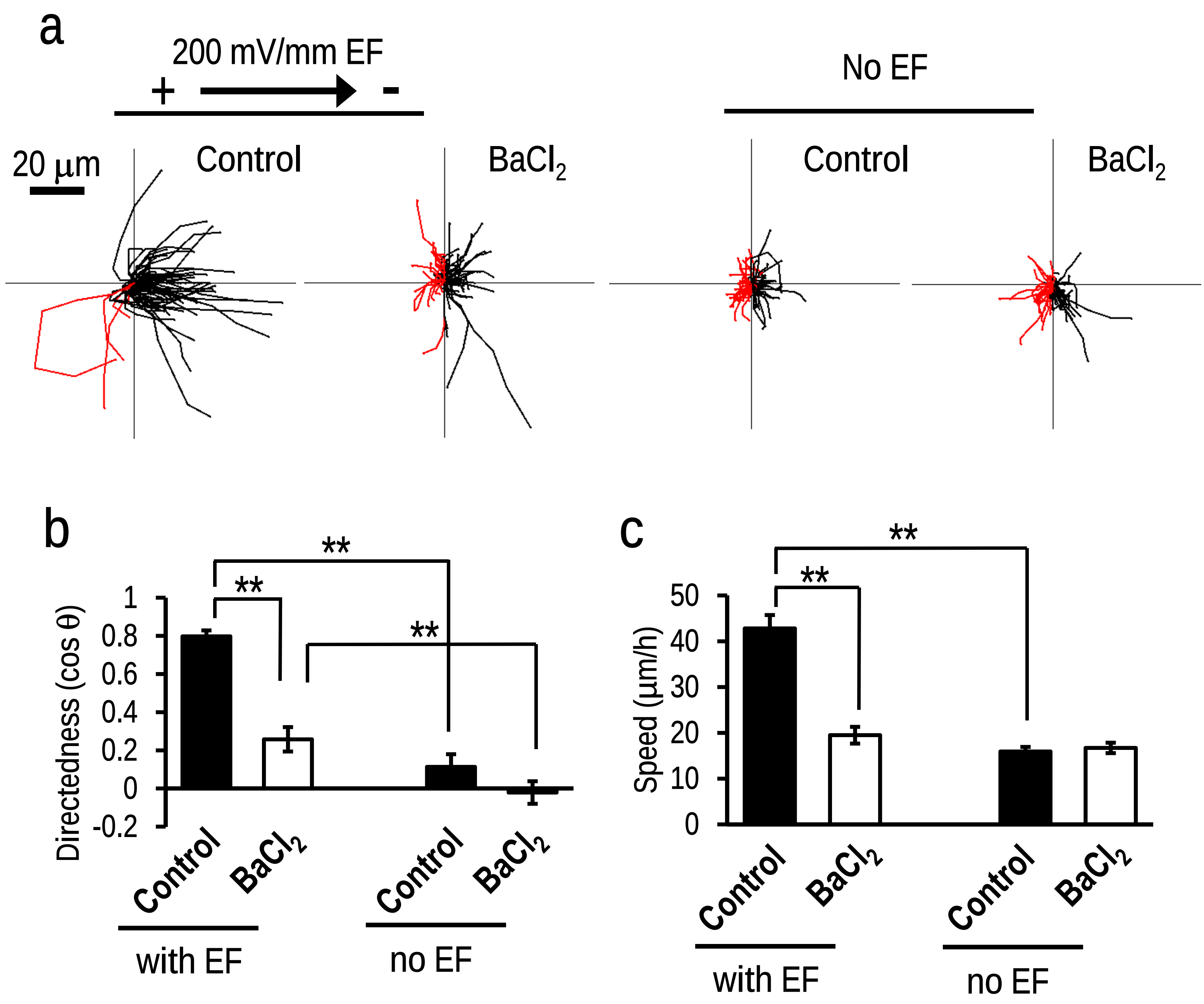

Supplementary Figure 5. Barium chloride at much lower concentration abolished galvanotaxis.

(a) Cells treated with $BaCl_2$ lost galvanotaxis. Black lines indicate trajectories of cells migrate toward cathode side. Red lines indicate trajectories of cells migrate toward anode side.

(b) Directedness values (cos θ) confirm loss of directionality.

(c) $BaCl_2$ treatment significantly inhibited migration speed.

n=100 cells for each group, confirmed in one other replicate.

$BaCl_2$ used at 100 μM. EF = 200 mV mm^{-1}. Statistical analysis was performed by the Student's *t*-test. Data represented as mean ± SEM. **, p<0.01.

Supplementary Figure 6

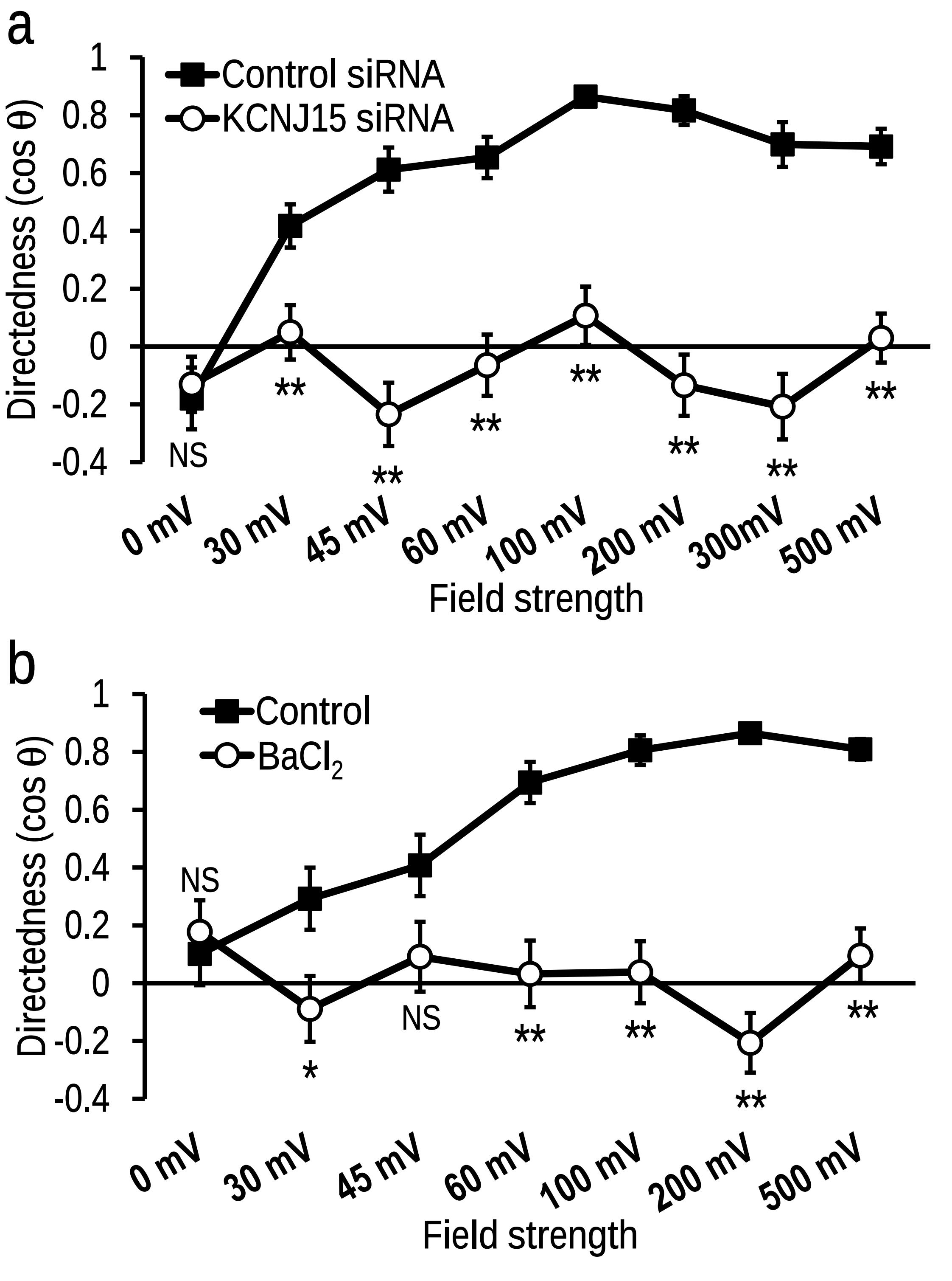

Supplementary Figure 6. KCNJ15 knockdown and $BaCl_2$ treatment abolished galvanotaxis in all voltage range tested.
(a, b) KCNJ15 knockdown and $BaCl_2$ (500 μM) treatment completely abolished galvanotaxis at all field strength tested (0-500 mV mm^{-1}). Statistical analysis was performed by the Student's *t*-test. Data represented as mean ± SEM. *, p<0.05. **, p<0.01. When the error bars are not seen, the bars are smaller than the symbols. n=40 cells for each group.

Supplementary Figure 7

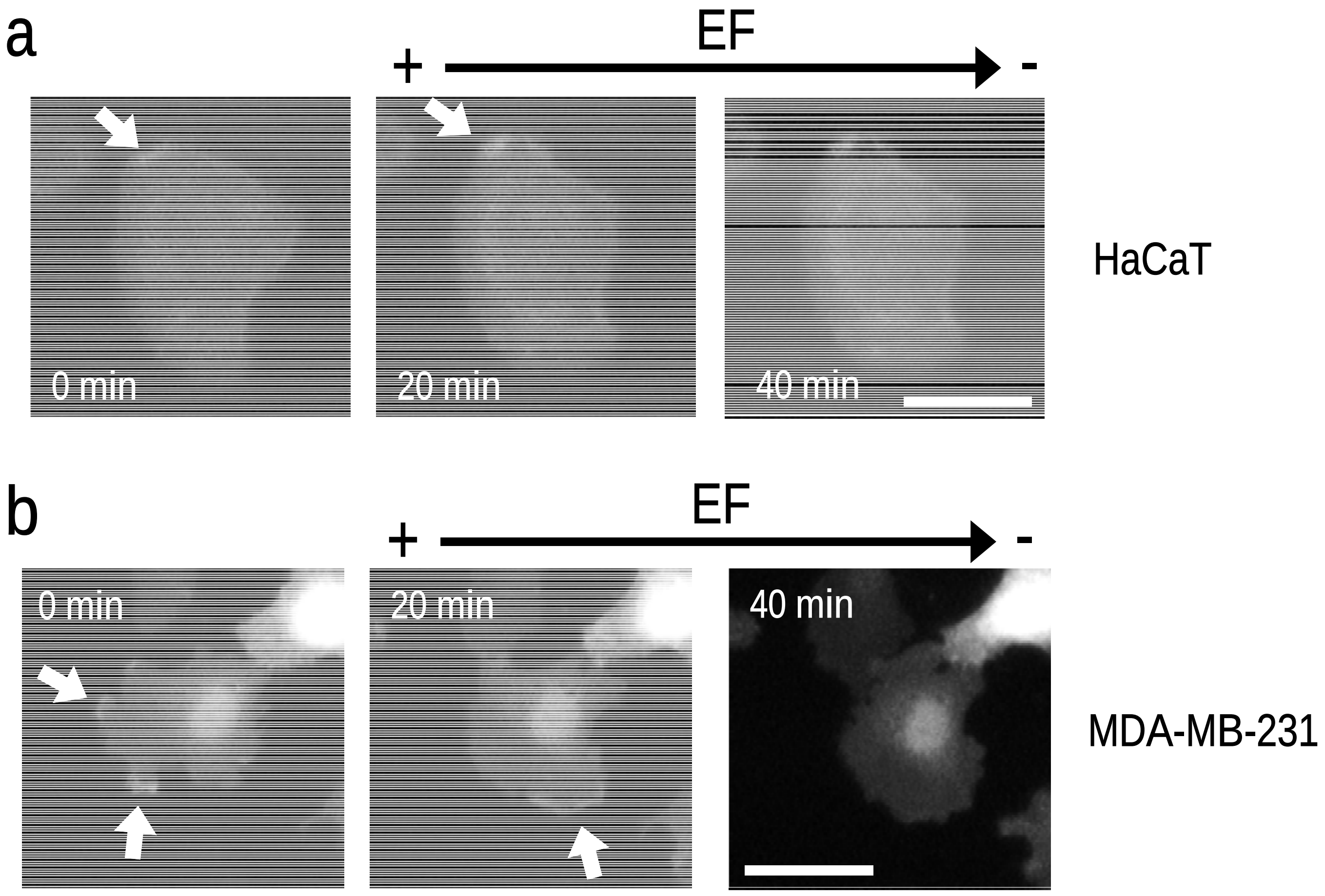

Supplementary Figure 7. No obvious polarization of PIP_3 in HaCaT and MDA-MB-231 cells.

HaCaT cells (**a**) and MDA-MB-231 (**b**) cells were transfected with pcDNA3-Akt-PH-EGFP plasmid DNA. Fluorescence of Akt-PH-EGFP was recorded by fluorescence microscope. Random and temporary PIP_3 accumulation was observed in certain part of the cells (arrows), even without EF application. Scale bar, 50 μm.

Supplementary Figure 8

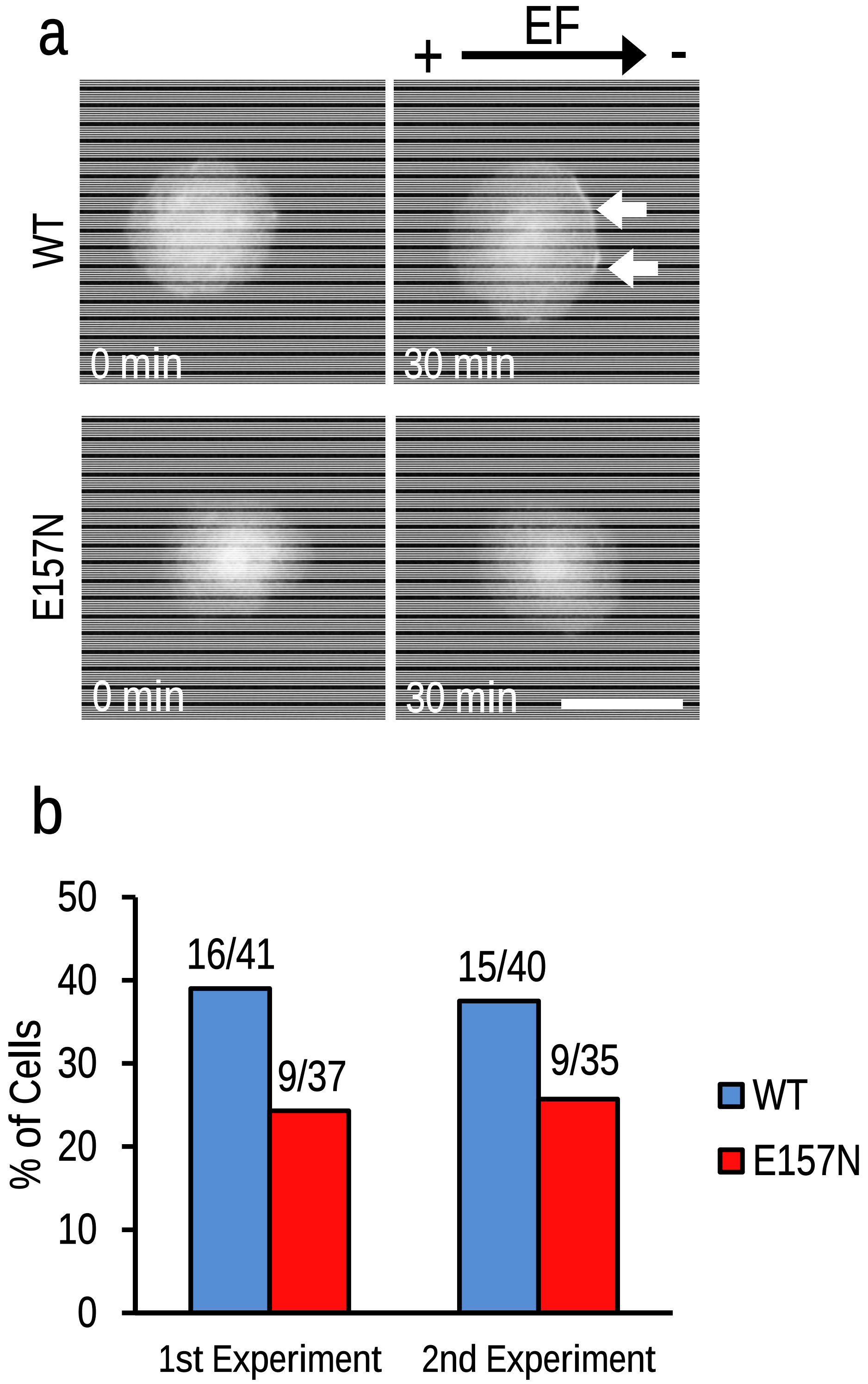

Supplementary Figure 8. Polyamine-binding defective mutant of Kir4.2 inhibited PIP_3 polarization to cathode facing side.

hTCEpi cells were infected with recombinant lentivirus to express WT or E157N Kir4.2, then transiently transfected with the expression construct of Akt-PH-EGFP. **(a)** Representative image of Akt-PH-EGFP localization in WT and E157N expressing hTCEpi cells. Arrow indicates cathodal polarization of PIP_3 (Akt-PH-EGFP). Scale bar, 50 μm. **(b)** Percentage of the cells with cathodal PIP_3 polarization within 30 min after EF application. Numbers above bar graph represent the number of the cells with cathodal PIP_3 polarization within 30 min after EF application / total number of cells counted in independent experiment.

Supplementary Figure 9

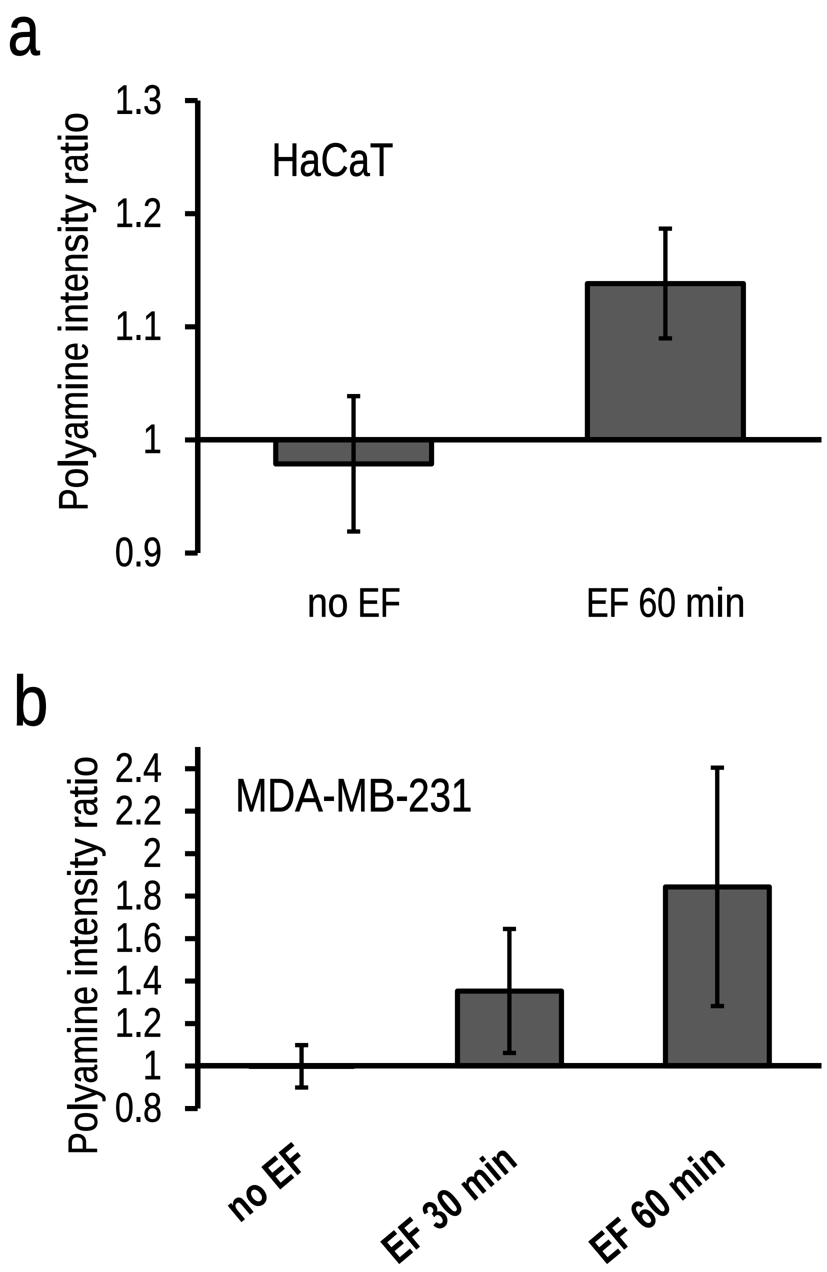

Supplementary Figure 9. Polyamines accumulated at cathode facing side in HaCaT and MDA-MB-231 cells.
HaCaT cells (**a**) or MDA-MB-231 cells (**b**) were exposed to an EF for 0, 30 or 60min, then fixed. Polyamines were stained and quantified as described in the legends for Figure 5i and j.

Supplementary Figure 10

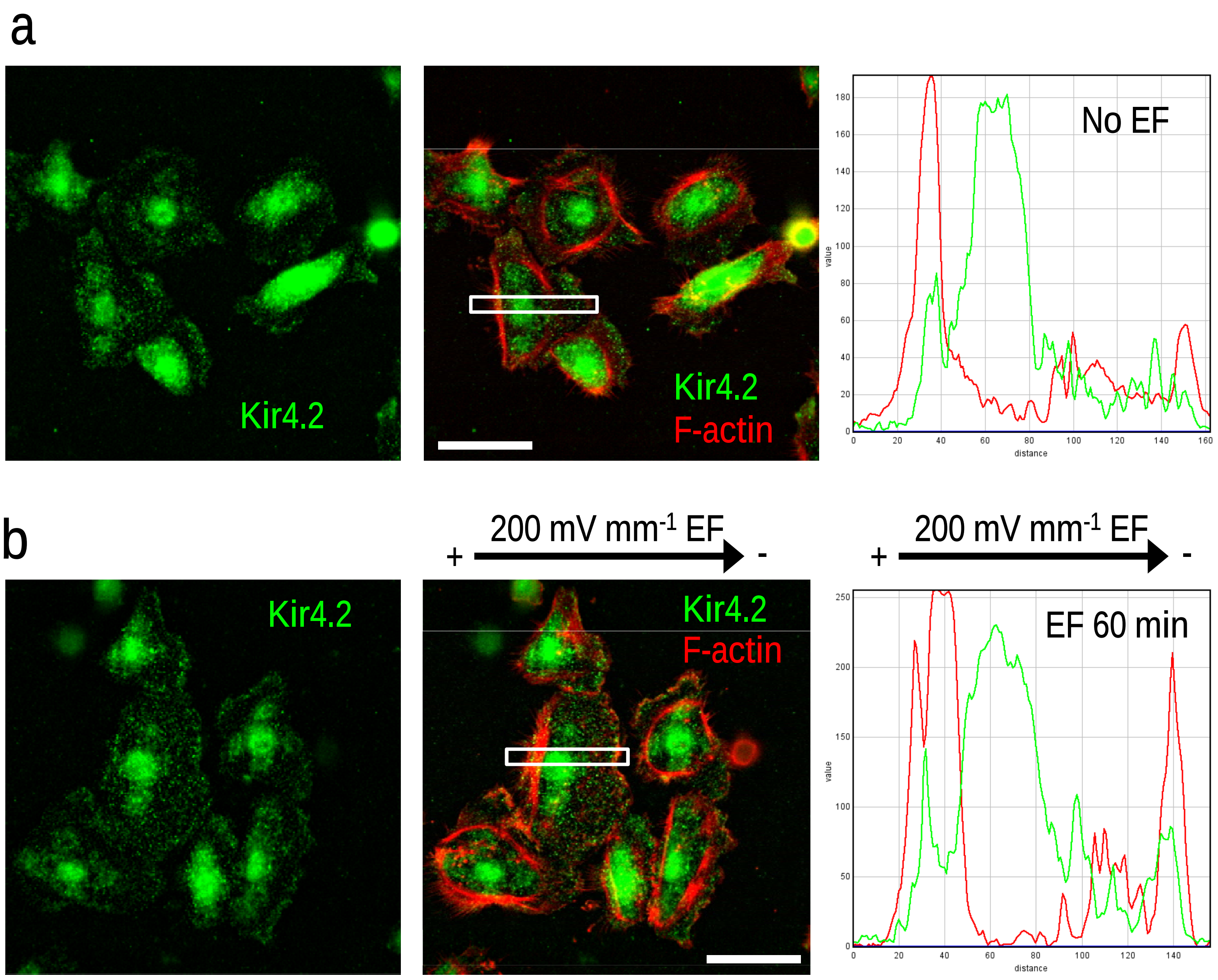

Supplementary Figure 10. Expression and localization of KCNJ15/Kir4.2 in hTCEpi cells. hTCEpi cells were exposed to an EF (200 mV mm^{-1}) for 0 or 60 min, then fixed with paraformaldehyde. Kir4.2 (green) and F-actin (red) was visualized with anti-Kir4.2 polyclonal antibody and Alexa555-conjugated phalloidin, respectively. Fluorescent intensities of white boxed area (20 pixel width line) were measured and visualized using RGB Line Profile function of ImageJ. Scale bar, 50 μm.

(a) no EF (0 min). **(b)** EF 60 min.

Supplementary Figure 11

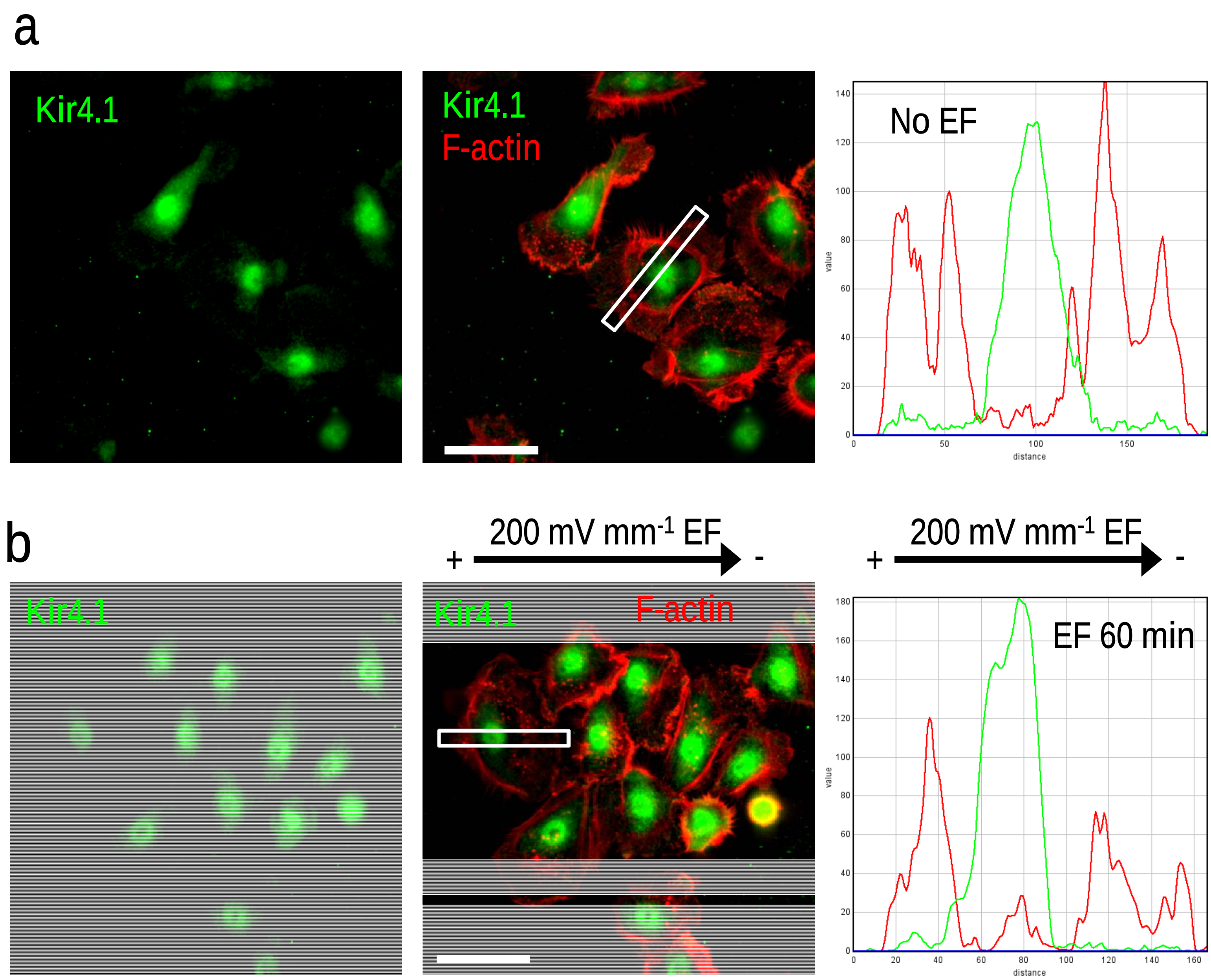

Supplementary Figure 11. Expression and localization of KCNJ10/Kir4.1 in hTCEpi cells. hTCEpi cells were exposed to an EF (200 mV mm^{-1}) for 0 or 60 min, then fixed with paraformaldehyde. Kir4.1 (green) and F-actin (red) was visualized with anti-Kir4.1 polyclonal antibody and Alexa555-conjugated phalloidin, respectively. Fluorescent intensities of white boxed area (20 pixel width line) were measured and visualized using RGB Line Profile function of ImageJ. Scale bar, 50 μm.
(a) no EF (0 min). **(b)** EF 60 min.

Supplementary Figure 12

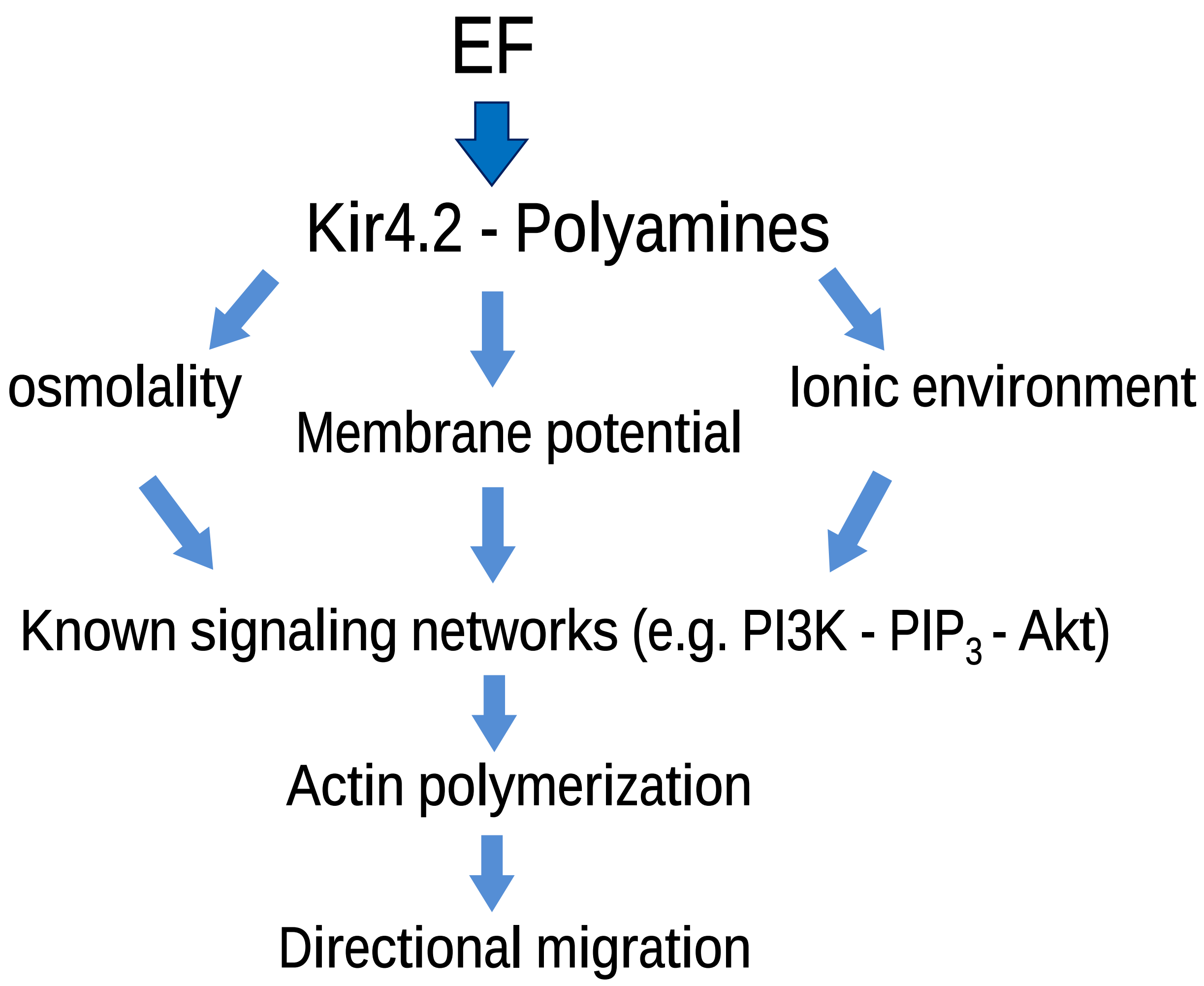

Supplementary Figure 12. Proposed model for polyamines and Kir4.2-mediated EF sensing.
Proposed model for polyamines and Kir4.2 in EF sensing. EF affect the interaction between polyamines and Kir4.2 leading the cell to sense extracellular EF and response ensues.

Supplementary Figure 13
Full size blot

Fig. 2a

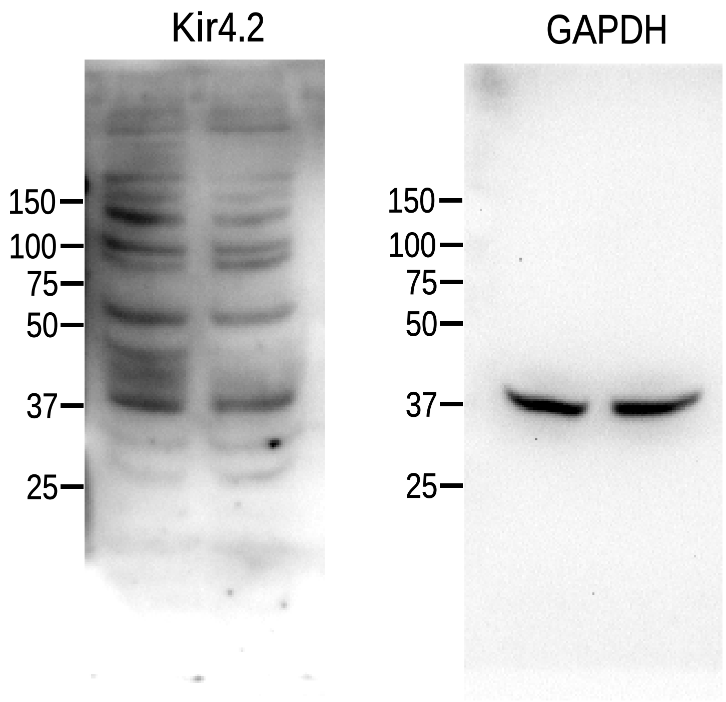

Fig. 5f

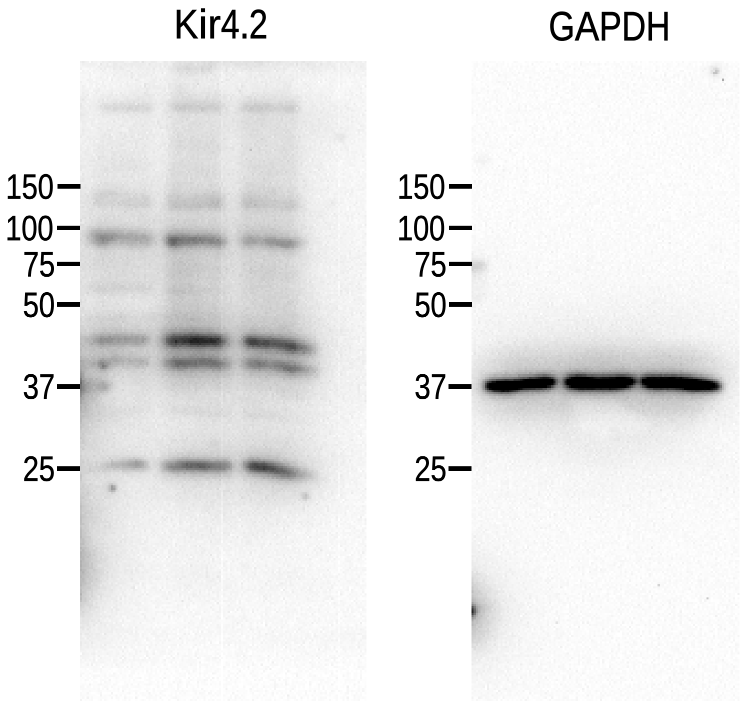

Supplementary Table 1. Complete abolishment of galvanotaxis only happened after KCNJ15 knockdown.

Gene (Protein)	Directedness (cos θ)
Control	0.635 ± 0.000819
KCNJ1 (Kir1.1)	0.745 ± 0.0576 (NS)
KCNJ2 (Kir2.1)	0.837 ± 0.0267 (NS)
KCNJ3 (Kir3.1)	0.652 ± 0.0708 (NS)
KCNJ4 (Kir2.3)	0.656 ± 0.0737 (NS)
KCNJ5 (Kir3.4)	0.873 ± 0.0316 (NS)
KCNJ6 (Kir3.2)	0.783 ± 0.0519 (NS)
KCNJ8 (Kir6.1)	0.747 ± 0.0632 (NS)
KCNJ9 (Kir3.3)	0.672 ± 0.0800 (NS)
KCNJ10 (Kir4.1)	0.692 ± 0.0880 (NS)
KCNJ11 (Kir6.2)	0.671 ± 0.0782 (NS)
KCNJ12 (Kir2.2)	0.714 ± 0.0719 (NS)
KCNJ13 (Kir7.1)	0.674 ± 0.0606 (NS)
KCNJ14 (Kir2.4)	0.535 ± 0.0906 (p<0.05)
KCNJ15 (Kir4.2)	0.115 ± 0.107 (p<0.01)
KCNJ16 (Kir5.1)	0.779 ± 0.0471 (NS)

Directedness (cos θ) of all of KCNJ (inward rectifying K^+ channel) knockdown cells were represented as mean ± SEM. Data are graphed as Fig. 1d. NS, no significance.

Supplementary Table 2. Front-Back signaling in an electric field is impaired in KCNJ15 knockdown cells.

	Percentage			
	Cathode	Random	Anode	Non
**Control**	**29.0**	**9.0**	**3.4**	**48.3**
**KCNJ15**	**11.5**	**11.5**	**0**	**76.9**

hTCEpi cells were transfected with siRNA and pcDNA3-Akt-PH-EGFP plasmid DNA. The cells were subjected to an EF (200 mV mm^{-1}), and fluorescence of Akt-PH-EGFP was recorded with a fluorescence microscope. Cells with strong cathode-facing side fluorescence accumulation (Cathode), anode-facing fluorescence accumulation (Anode), random area fluorescence accumulation (Random), and cells with no strong fluorescence accumulation in any area are scored (Non). Data were pooled from 26-40 cells from two independent experiments.

Supplementary Table 3. Front-Back signaling in an electric field is impaired in $BaCl_2$-treated cells.

	Percentage			
	**Cathode**	**Random**	**Anode**	**Non**
**Control**	**31.6**	**10.5**	**0**	**57.9**
**$BaCl_2$**	**5.0**	**5.0**	**7.5**	**82.5**

hTCEpi cells were transfected with pcDNA3-Akt-PH-EGFP plasmid DNA. The cells were subjected to an EF (200 mV mm^{-1}) in the presence or absence of $BaCl_2$ (500 μM), and fluorescence of Akt-PH-EGFP was recorded with a fluorescence microscope. Cells with strong cathode-facing side fluorescence accumulation (Cathode), anode-facing fluorescence accumulation (Anode), random area fluorescence accumulation (Random), and cells with no strong fluorescence accumulation in any area are scored (Non). Data were pooled from 26-40 cells from two independent experiments.
